# Dissecting antibiotic effects on the cell envelope using bacterial cytological profiling: a phenotypic analysis starter kit

**DOI:** 10.1128/spectrum.03275-23

**Published:** 2024-01-30

**Authors:** Ann-Britt Schäfer, Margareth Sidarta, Ireny Abdelmesseh Nekhala, Gabriela Marinho Righetto, Aysha Arshad, Michaela Wenzel

**Affiliations:** 1 Division of Chemical Biology, Department of Life Sciences, Chalmers University of Technology, Gothenburg, Sweden; 2 Center for Antibiotic Resistance Research in Gothenburg (CARe), Gothenburg, Sweden; Centre National de la Recherche Scientifique, Rue Michel Ange, Paris, France

**Keywords:** bacterial cytological profiling, antimicrobial agents, mechanism of action, fluorescence assays, valinomycin, vancomycin, nisin

## Abstract

Phenotypic analysis assays such as bacterial cytological profiling (BCP) have become increasingly popular for antibiotic mode of action analysis. A plethora of dyes, protein fusions, and reporter strains are available and have been used for this purpose, enabling both rapid mode of action categorization and in-depth analysis of antibiotic mechanisms. However, non-expert researchers may struggle choosing suitable assays and interpreting results. This is a particular problem for antibiotics that have multiple or complex targets, such as the bacterial cell envelope. Here, we set out to curate a minimal set of accessible and affordable phenotypic assays that allow distinction between membrane and cell wall targets, can identify dual-action inhibitors, and can be implemented in most research environments. To this end, we employed BCP, membrane potential, fluidity, and cell wall synthesis assays. To assess specificity and ease of interpretation, we tested three well-characterized and commercially available reference antibiotics: the potassium ionophore valinomycin, the lipid II-binding glycopeptide vancomycin, and the dual-action lantibiotic nisin, which binds lipid II and forms a membrane pore. Based on our experiments, we suggest a minimal set of BCP, a membrane-potentiometric probe, and fluorescent protein fusions to MinD and MreB as basic assay set and recommend complementing these assays with Laurdan-based fluidity measurements and a *PliaI* reporter fusion, where indicated. We believe that our results can provide guidance for researchers who wish to use phenotypic analysis for mode of action studies but do not possess the specialized equipment or expert knowledge to employ the full breadth of possible techniques.

**IMPORTANCE:**

Phenotypic analysis assays using specialized fluorescence fusions and dyes have become increasingly popular in antibiotic mode of action analysis. However, it can be difficult to implement these methods due to the need for specialized equipment and/or the complexity of bacterial cell biology and physiology, making the interpretation of results difficult for non-experts. This is especially problematic for compounds that have multiple or pleiotropic effects, such as inhibitors of the bacterial cell envelope. In order to make phenotypic analysis assays accessible to labs, whose primary expertise is not bacterial cell biology, or with limited equipment and resources, a set of simple and broadly accessible assays is needed that is easy to implement, execute, and interpret. Here, we have curated a set of assays and strains that does not need highly specialized equipment, can be performed in most labs, and is straightforward to interpret without knowing the intricacies of bacterial cell biology.

## INTRODUCTION

An antibiotic’s mode of action is decisive for how easily bacteria can develop resistance against it. Therefore, understanding antibiotic mechanisms is crucial to inform new drug design and to develop the next generations of antibiotics (1). Also, a new drug’s mechanism must typically be sufficiently characterized before it can be brought to the market. Thus, mode of action analysis is a pivotal part of the preclinical characterization of antibiotic candidates. A plethora of methodologies can be used for this purpose, differing considerably with respect to the detail they provide, their sensitivity, complexity, and accessibility, as well as the time and work effort required (2).

Over the years, it has become clear that *in vitro* assays with isolated cell components are not capturing the full complexity of the effects antibiotics have on bacteria. This is, for example, illustrated by daptomycin, which forms membrane pores in model membranes but not in bacterial cells (3–5). Thus, methods that can be applied to bacterial cultures are highly desirable and particularly powerful when combined with specific *in vitro* techniques, such as enzyme inhibition assays.

A method that has become increasingly popular for *in vivo* mode of action analysis is bacterial cytological profiling (BCP). BCP is a phenotypic analysis technique that in its original form makes use of phase contrast microscopy combined with fluorescent dyes that stain the bacterial nucleoid and cell membrane (6). It can be applied to diverse bacterial species (7–9) and is typically used for rapid mode of action classification (6, 10, 11) but can also be used to identify new antibiotic targets (12) and to determine antibiotic susceptibility (13). Variations include time-resolved and high-resolution BCP, which provide additional temporal and spatial information (14, 15). The term BCP has also been more broadly used to describe phenotypic analyses that are based on advanced cell biological methods. As such, it has been applied to extensive mode of action studies that employ a combination of several specialized fluorescent dyes and protein fusions (16–19). In this study, we use the term BCP specifically for a phenotypic analysis assay that combines fluorescent membrane and DNA staining with a reporter for membrane pores and phase contrast, in accordance with the originally reported method (6), and “phenotypic analysis” as an umbrella term that includes our definition of BCP as well as other cell biological techniques.

Phenotypic analysis offers the full range from fast high-throughput mode of action categorization to incredibly detailed in-depth analysis of antibiotic mechanisms. However, the plethora of possible dyes, strains, and assays can make it difficult to choose which specific phenotypic experiments are the most suited for a given purpose, especially when bacterial cell biology is not the main expertise of the experimenter. Bacterial cell physiology is complex, and many cellular processes are intimately interconnected, e.g., by co-dependent regulation or metabolic flux. Moreover, phenotypes can differ considerably, depending on the growth conditions, leading to varying results (20). These factors can make the interpretation of phenotypic analysis data difficult (21).

Another challenge is posed by antibiotics with multiple mechanisms of action or pleiotropic downstream effects. For a long time, drug development has focused on single protein targets, yet the antibiotic resistance crisis has led to a re-evaluation of previously neglected multitarget molecules as well as the deliberate design of multifunctional compounds and hybrid molecules (22). One example for this is telavancin, a vancomycin derivative that, like its parent compound, inhibits cell wall synthesis by binding to the peptidoglycan precursor lipid II but possesses an additional lipid tail that allows it to interact with and depolarize the cell membrane (23). Mode of action analysis of such multifunctional antimicrobials can be challenging, especially for compounds that were not deliberately designed as such like telavancin but have inherent and unknown multifunctional properties, for example, certain antimicrobial peptides or aminoglycosides (24–26). It can become even more difficult when analyzing compounds that target the cell envelope, since the cell wall and membrane(s) are closely connected, both structurally and functionally. Yet, cell wall synthesis is still the most successful and common clinical antibiotic target, and the cytoplasmic membrane has moved more and more into the spotlight of antibacterial drug discovery (27, 28).

**Fig 1:**
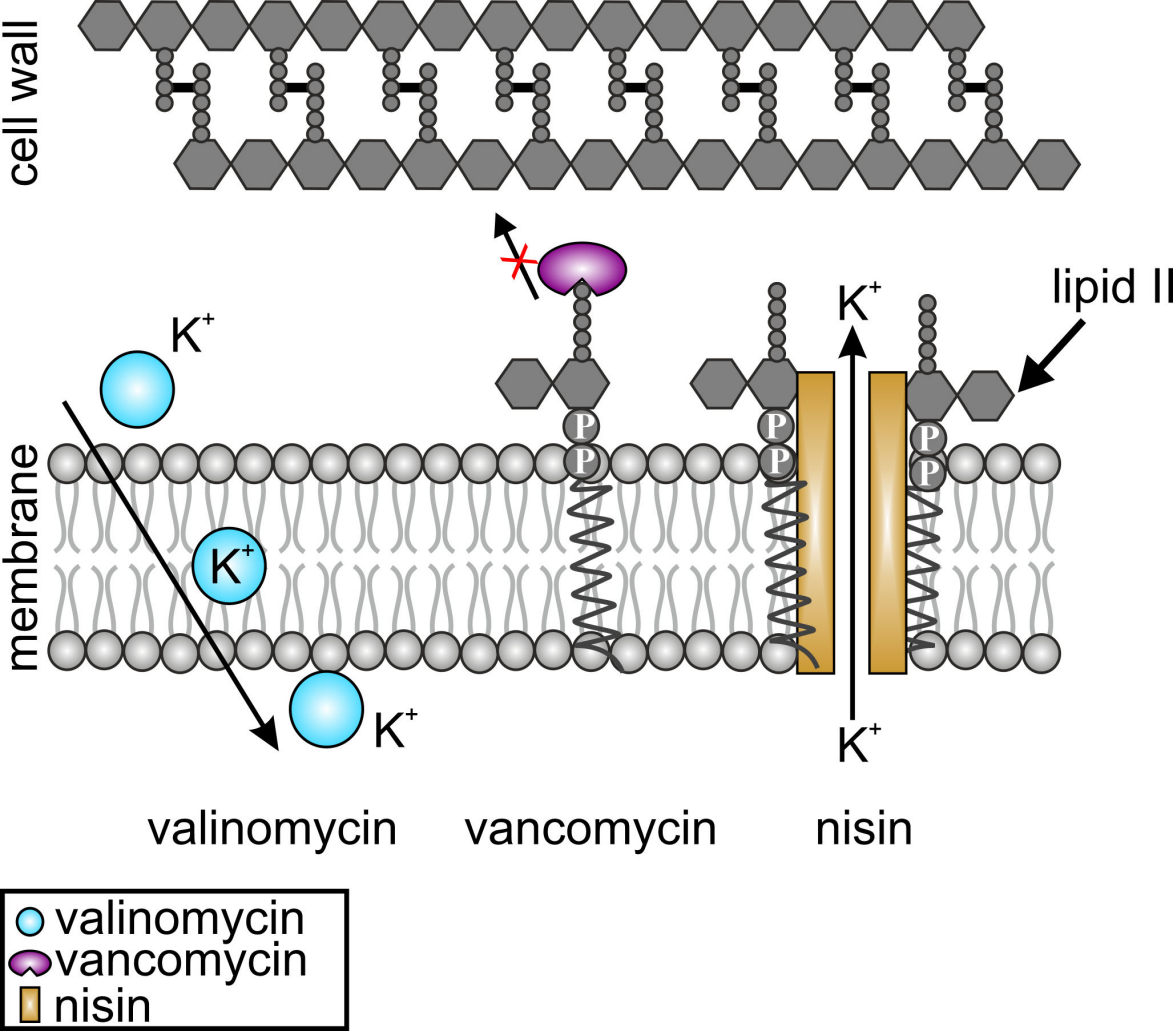
Mechanisms of action of valinomycin (blue), vancomycin (purple), and nisin (yellow). Valinomycin is a potassium carrier ionophore that depolarizes the cell membrane (29). Vancomycin binds to the D-Ala-D-Ala residue of lipid II, inhibiting its incorporation into the peptidoglycan cell wall (30). Nisin is a dual-action antibiotic that binds to the sugar-phosphate moiety of lipid II and uses it as a docking molecule to form a transmembrane pore (31).

Here, we set out to curate a robust and accessible set of assays, based on BCP and other phenotypic analysis techniques, that is suitable to distinguish between membrane and cell wall effects and capable of identifying dual-action inhibitors. To this end, we used three well-characterized and commercially available antibiotics: valinomycin, vancomycin, and nisin (Fig. 1; Table S1).

Valinomycin is a cyclic dodecadepsipeptide that acts as a potassium carrier ionophore and disturbs the electrochemical gradient, leading to membrane depolarization (29). It has no known additional effects, making it a good representative for a single-mechanism membrane antibiotic. Vancomycin is a glycopeptide antibiotic that binds to the D-ala-D-ala moiety of the membrane-bound peptidoglycan precursor lipid II and prevents its incorporation into the cell wall (30). Despite binding to a membrane-bound target, vancomycin does not disturb the cell membrane itself and is thus a good representative of a specific cell wall synthesis inhibitor (32). Nisin is a class A lantibiotic with a dual mechanism of action. It binds to the sugar-pyrophosphate group of lipid II, inhibiting cell wall synthesis in a similar manner to vancomycin. Additionally, it uses lipid II as a docking molecule to form a large transmembrane pore, resulting in dissipation of the membrane potential and profound intracellular content leakage (31). It is thus a good example for a dual inhibitor of cell wall and membrane functions. Comparing nisin with valinomycin additionally allows distinction between membrane depolarization and large-scale pore formation.

To assess the specificity of selected assays, we further included 10 comparator compounds with different mechanisms (Table S2). This selection comprised two additional membrane-active compounds, the Na^+^/K^+^ channel ionophore gramicidin (gramicidin D = a mix of gramicidin A–C) and the H^+^ carrier ionophore carbonyl cyanide m-chlorophenyl hydrazone (CCCP), and two additional cell wall synthesis inhibitors: D-cycloserine, inhibiting both alanine racemase and D-ala-D-ala ligase, and tunicamycin, inhibiting the lipid I synthase MraY. Additionally, we selected six compounds with targets unrelated to the cell envelope, namely, the gyrase/topoisomerase IV inhibitor ciprofloxacin, the RNA polymerase inhibitor rifampicin, the ribosome inhibitors tetracycline, kanamycin, and chloramphenicol, and the pro-drug nitrofurantoin, which generates reactive species that damage cellular macromolecules (33).

We examined the effects of these antibiotics in different phenotypic assays, focusing on techniques that are easy to implement and execute, do not require highly specialized instrumentation, and can be interpreted without excessive knowledge of bacterial cell biology. To this end, we evaluated a slightly modified BCP assay as well as methods to assess membrane potential, membrane fluidity, and cell wall synthesis based on our previous experiences with in-depth mode of action analysis of cell envelope-targeting antibiotics (2, 3, 16–19, 33, 34). We chose *Bacillus subtilis* as a model since it is already a popular model for antibiotic mode of action studies (19, 35–39), can be used in biosafety level 1 labs, is easy and inexpensive to handle, and is susceptible to most antibiotics.

## RESULTS AND DISCUSSION

### Antibiotic concentrations for phenotypic experiments

We first determined suitable concentrations for antibiotic stress experiments. This is a crucial step that ensures bacteria are sufficiently inhibited to show a clear phenotype, yet not fully inhibited or undergoing cell lysis, which would lead to unspecific observations caused by cell death and disintegration. To this end, minimal inhibitory concentrations (MICs) were determined followed by acute shock experiments to identify a concentration leading to a 50%–70% reduction of bacterial growth in exponential growth phase (Table S1; Fig. S1 and S2). The selected concentrations were 10-µg/mL valinomycin, 0.5-µg/mL vancomycin, and 0.8-µg/mL nisin (see Table S2 for concentrations of comparator compounds). Refer to Text S1 for detailed explanations and pitfalls of selecting antibiotic concentrations, growth conditions, and fluorescence dyes for phenotypic analysis assays.

### Bacterial cytological profiling

**Fig 2:**
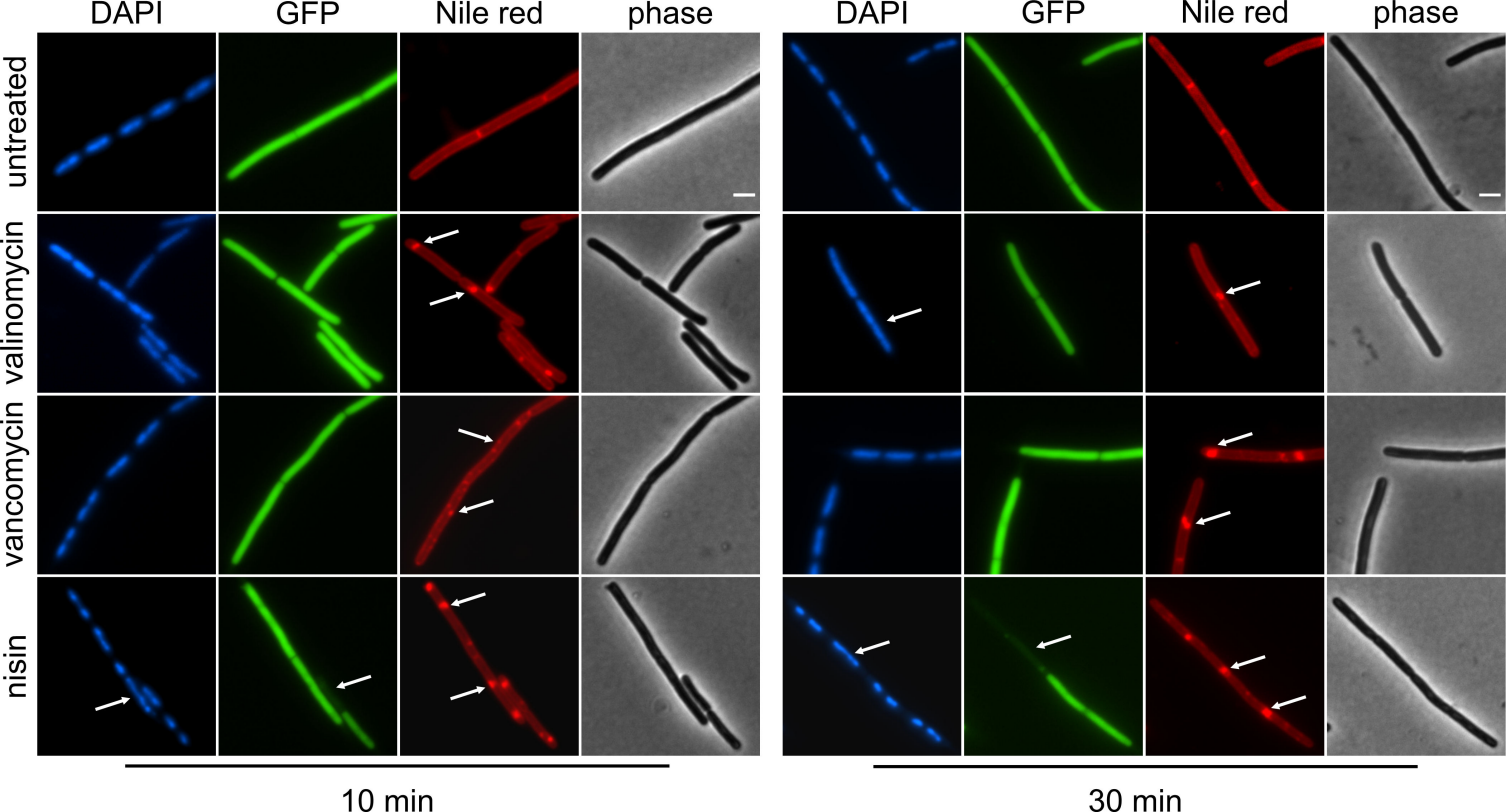
Bacterial cytological profiling of *B. subtilis* bSS82 (*PrpsD-gfp*) treated with antibiotics for 10 and 30 min. DNA was stained with DAPI; cytosolic GFP was expressed from the constitutive *PrpsD* promoter, and membranes were stained with Nile red. Morphological changes are marked with arrows. Scale bar 2 µm.

We started out with a slightly modified version of the original BCP assay. To this end, we used a *B. subtilis* strain that expresses cytosolic green-fluorescent protein (GFP) from a strong constitutive promoter, enabling assessment of pore formation using the loss of intracellular GFP signal as readout. DNA was stained with 4′,6-diamidino-2-phenylindole (DAPI) and cell membranes with Nile red (for guidelines on the selection of dyes, see Text S1; Fig. S3 through S7). Phase contrast, while not essential for this assay, allows easier assessment of morphological changes than bright-field microscopy and additionally serves as internal control for cell lysis, which is clearly visible as loss of phase density (Fig. S8). BCP results for valinomycin, vancomycin, and nisin are shown in Fig. 2 and summarized in Table 1. See Fig. S9 and S10 for quantification of BCP images and refer to Text S2 for further elaboration on and common pitfalls of quantitative image analysis.

**TABLE 1:** Results overview of phenotypic assays^*a*^

		Valinomycin	Vancomycin	Nisin
BCP	DAPI	**+**	**−**	**+**
	Nile red	**+**	**+**	**+**
	GFP	−	**−**	**+**
Membrane	MinD	**+**	**−**	**+**
	DiSC_3_5	**+**	**−**	**+**
	Laurdan	**+**	**−**	**+**
	DiIC12	**+**	**+**	**+**
Cell wall	MurG	**+**	**+**	**+**
	MraY	**−**	**−**	**+**
	MreB localization	**+**	**+**	**+**
	MreB mobility	**−**	**+**	**+**
	*PliaI*	**−**	(**+**)	**+**

^*a*^ Positive results, defined as differing from the untreated control, are indicated with +, while negative results, defined as not visibly different from the untreated control, are indicated with −. (+) indicates a slightly positive effect.

DAPI staining revealed opposing effects for nisin and valinomycin, while vancomycin showed no difference to the untreated control. Thus, nisin treatment led to clear nucleoid condensation already after 10 min (Fig. 2; Fig. S9). Similar observations have been made with other pore-forming compounds (17), suggesting that leakage of intracellular contents and corresponding shrinking of the protoplast underlie this effect. In contrast, valinomycin caused relaxation of the nucleoid after 30 min. A similar effect was observed with the ionophores CCCP and gramicidin (Fig. S11), suggesting that this relaxation is a downstream effect of membrane depolarization. Indeed, it has been shown that ionophores can cause DNA fragmentation in mammalian cells (40). In both cases, DNA packing defects occur as non-specific downstream effects of target inhibition, which can be observed with a variety of compounds with diverse mechanisms of action (6). For example, nucleoid condensation was also observed with tetracycline and relaxation with rifampicin and nitrofurantoin (Fig. S11).

While loss of intracellular GFP, indicating leakage of molecules as large as 27 kDa, was only observed for nisin, as was expected, all three compounds showed clear effects on the membrane stain, visible as brightly fluorescent foci (Fig. 2; Fig. S10). Such foci can be caused by depolarization, accumulation of lipid II, or membrane phase separation (3, 9, 17, 41) and were accordingly also observed with gramicidin, CCCP, tunicamycin, and D-cycloserine. However, they also appeared in cells treated with tetracycline, chloramphenicol, and nitrofurantoin (Fig. S11). Tetracycline has been shown to have secondary effects on the cell membrane (42), and nitrofurantoin may cause lipid peroxidation (33), possibly explaining these effects. Yet, why chloramphenicol elicits Nile red foci is unclear.

Taken together, several compounds with unrelated mechanisms displayed secondary or downstream effects on the nucleoid and cell membrane. This is underlined by the patterns elicited by the chosen comparator compounds (Table 1; Table S3). For example, chloramphenicol and kanamycin elicited a similar phenotype as vancomycin, tunicamycin, and D-cycloserine, while nitrofurantoin produced the same pattern as the tested ionophores.

BCP, as defined here, correctly and specifically identified nisin as a pore former but could not unambiguously identify and distinguish between membrane and cell wall-related mechanisms of action. While the method has been successfully used for many other mechanistic classes (6), distinguishing between different cell envelope-related mechanisms is not its forte. It can certainly be a good starting point for generating a first hypothesis on a new compound’s mechanism but requires additional follow-up experiments. The advantage of BCP is that the dyes and fusions used can be adjusted to the project’s needs. Thus, we used intracellular GFP to identify a pore former. Yet, it is possible to use different GFP fusions, e.g., to FtsZ for identification of cell division inhibitors (35), making the assay extremely versatile. Other than DAPI and Nile red, other dyes with different spectral properties may be used to visualize the DNA and cell membrane, allowing the use of, e.g., protein fusions to red-fluorescent protein (RFP) or cyan-fluorescent protein (CFP). While DNA dyes are usually toxic and Nile red displays phototoxicity, non-toxic membrane dyes such as FM5-95 allow the use of time-lapse microscopy to follow antibiotic effects over time (2) (see also Text S1; Fig. S3 and S4).

### Membrane depolarization

Most membrane-active antimicrobial compounds affect the membrane potential, and assessing depolarization often gives a good indication of whether a compound targets the cell membrane or not. Here, we employed two easy-to-implement depolarization assays: DiSC_3_(5) fluorescence and MinD localization.

**Fig 3:**
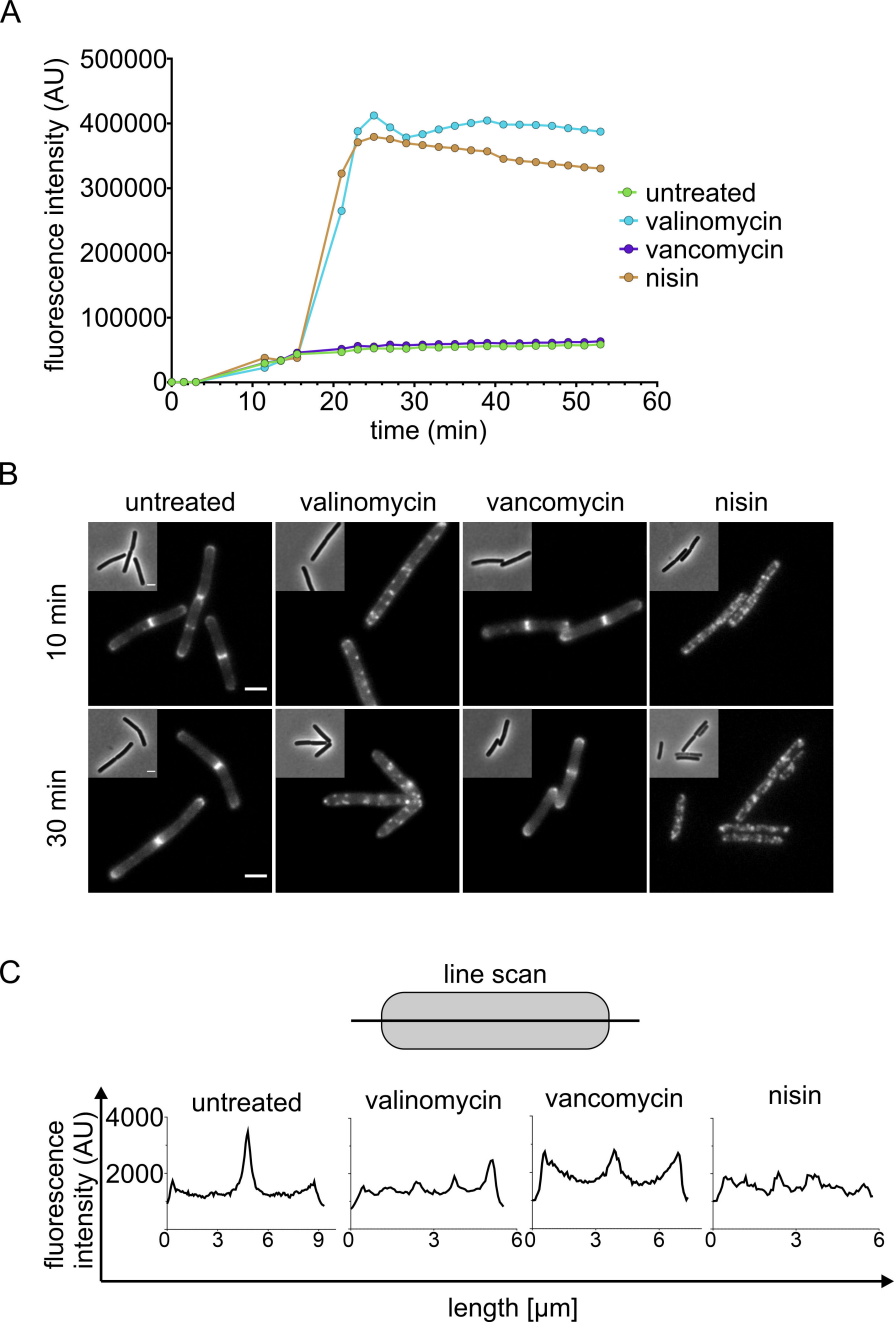
Effects of valinomycin, vancomycin, and nisin on the membrane potential. (**A**) Spectroscopic membrane potential measurements of *B. subtilis* 168CA (wild type) with the fluorescence dye DiSC_3_(5). (**B**) Localization of the cell division regulation protein MinD after 10 and 30 min of antibiotic treatment [strain *B. subtilis* TB35 (*Pxyl-gfp-minD*)]. (**C**) Fluorescence profiles of cells shown in panel **B**, measured along the lateral cell axis pertaining to 30 min of antibiotic treatment.

DiSC_3_(5) is a self-quenching, voltage-sensitive dye that accumulates in polarized membranes and is released upon depolarization, resulting in a fluorescence increase due to de-quenching (21). Depolarization was clearly visible with both valinomycin and nisin, while vancomycin had no effect (Fig. 3A), providing a much clearer and unambiguous readout than BCP alone. However, some compounds are not compatible with the DiSC_3_(5) dye, either due to spectral interference or an interaction with the dye itself. An alternative to DiSC_3_(5) is the fluorescence dye DiBAC_4_(3), which is excluded from polarized cells but accumulates intracellularly when the membrane potential is dissipated. In contrast to DiSC_3_(5), which is a positively charged, far-red fluorescent dye, DiBAC_4_(3) is a negatively charged, green-fluorescent dye (21). Thus, DiBAC_4_(3) is a viable alternative in many cases, where an antimicrobial compound interferes with DiSC_3_(5), and vice versa. Another membrane potential reporter is tetraphenylphosphonium ion, yet these measurements are based on radioactivity, which makes the method less accessible (43).

Due to this dye interference issue, it can be beneficial to have a dye-independent membrane depolarization assay at hand (21). One tool that has been employed for this purpose is a GFP fusion to the cell division regulation protein MinD (21, 32, 44–47). MinD is a peripheral membrane protein that binds to the lipid bilayer through an amphipathic α-helix motif that requires a membrane potential to bind to the cell membrane (44). In cells with an intact membrane potential, MinD localizes at mid-cell and at the cell poles. This regular pattern becomes spotty in depolarized cells and can be accompanied by a loss of membrane binding (19, 21, 44). This effect was very well visible in valinomycin- and nisin-treated cells and absent in vancomycin-treated samples (Fig. 3B and C), matching the DiSC_3_(5) results.

MinD is the most commonly used protein fusion reporter for depolarization. It is relatively robust and shows a clear localization change upon depolarization. Yet, it is sensitive to protein expression levels as its overexpression inhibits cell division, leading to strongly elongated cells with aberrant localization (Fig. S6). MreB can be used as an alternative, yet its native spotty pattern can make it difficult for the untrained eye to identify depolarization-induced clusters (41, 44). Another alternative is the cell division protein FtsA, which normally localizes at the septum and loses its membrane-binding upon depolarization, providing an unambiguous readout (44). Unlike fluorescent dyes, protein fusions do not have the issue of compound interference, yet they are not always highly specific for depolarization. For example, the antimicrobial peptide cWFW causes large-scale phase separation that promotes displacement of the membrane-binding domain of MinD into clusters yet has only a minor, transient effect on the membrane potential, which by itself is not sufficient to delocalize MinD (34). Further, MinD is also affected by non-depolarizing compounds like tetracycline and nitrofurantoin (Fig. S12), possibly due to membrane fluidity changes (Fig. S13). Due to these limitations, it is advisable to use a dye-based assay together with a fusion protein reporter.

### Membrane fluidity

Not all membrane-targeting antibiotics cause depolarization and such mechanisms may be missed when relying on potential measurements alone (34). Several studies have shown that membrane fluidity is another crucial factor for the mechanisms of membrane-active antibiotics that may or may not occur together with depolarization (3, 16, 17, 34). For these reasons, assessment of membrane fluidity can be useful in addition to depolarization assays.

**Fig 4:**
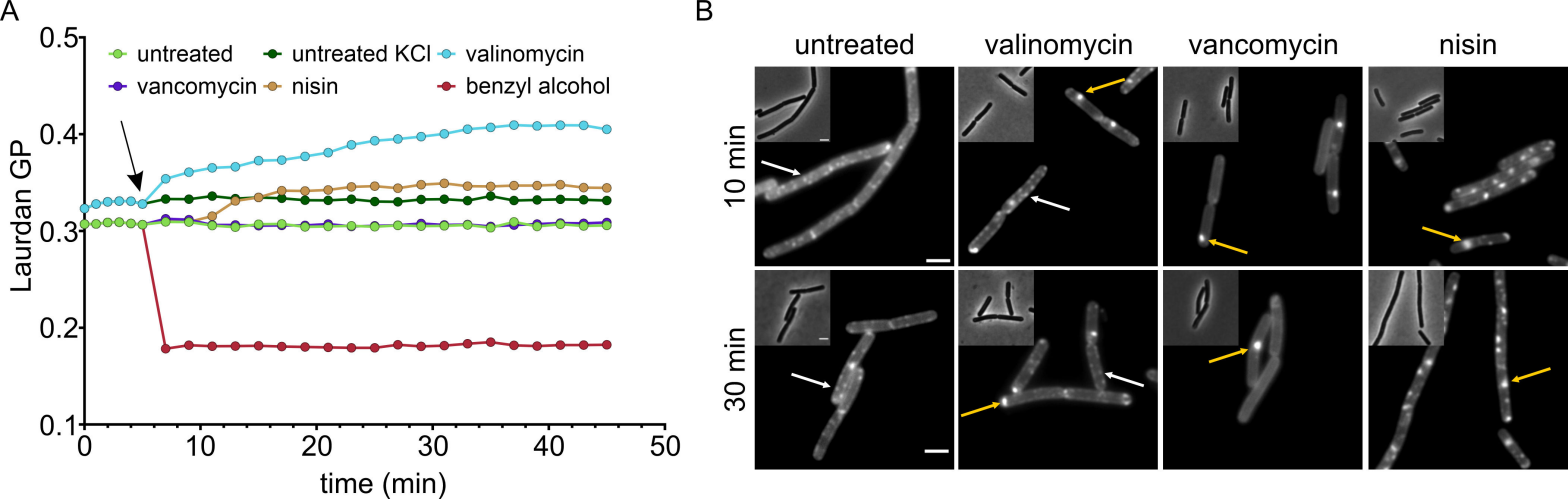
Effects of valinomycin, vancomycin, and nisin on membrane fluidity. (**A**) Overall membrane fluidity of *B. subtilis* 168CA (wild type) measured by Laurdan generalized polarization (GP). Black arrow indicates the addition of antibiotics to *B. subtilis*. Note the different GP values for untreated cells grown in standard Mueller-Hinton broth (MHB) and KCl-MHB. (**B**) Visualization of fluid membrane microdomains (regions of increased fluidity) in *B. subtilis* 168CA (wild type) with the fluidity-sensitive fluorescence dye DiIC12. Exponentially growing *B. subtilis* cells exhibit a spotty DiIC12 pattern (white arrows) (17, 41). Large DiIC12 clusters are indicated with yellow arrows.

Laurdan is a fluorescent dye that inserts into membranes and exhibits a shift in its emission peak, depending on the amount of water molecules in its proximity. This shift can be used as an indication of lipid head group and fatty acyl chain spreading and expressed as generalized polarization value, providing a readout for membrane fluidity (48, 49). As depicted in Fig. 4A, both valinomycin and nisin caused rigidification of the cell membrane, while vancomycin had no effect on membrane fluidity. While the effect of valinomycin was immediate, rigidification by nisin seemed to be delayed by 6–8 min, which could possibly reflect its two-staged mechanism of action.

Laurdan is a cheap, commercially available fluidity sensor that has been successfully employed for antibiotic mode of action studies in the past (3, 16–18, 34) and for which detailed experimental protocols have been published (48, 49) (see also Text S5). Laurdan can be used to spectroscopically assess overall membrane fluidity, yet its spectral properties are not compatible with standard filter sets, limiting its accessibility for less specialized labs (48, 49). An alternative to Laurdan is the fluorescence anisotropy probe 1,6‐diphenyl‐1,3,5‐hexatriene, which delivers comparable results but requires a spectrometer equipped with a polarizer (50). A dye-independent measure of membrane fluidity is fluorescence recovery after photobleaching, which assesses diffusion of protein fusions or dyes in the membrane (51–53), yet this requires a suitably equipped confocal fluorescence microscope and is thus not accessible to many researchers.

While Laurdan can in principle be visualized under a fluorescence microscope (3, 16, 17, 48, 49), this requires custom filter sets and a very high-quality objective. DiIC12 is an alternative commercially available dye that can be used to visualize membrane fluidity. It is best visualized using a Cy3 filter, but standard RFP filters can also be used (48). The dye preferably inserts into more fluid membrane regions due to its short hydrocarbon tail and thus stains fluid membrane domains (48, 49). DiIC12 is a qualitative measure of membrane fluidity but is very sensitive and allows visualization of fluid membrane microdomains that usually escape detection by other membrane dyes (2). In rod-shaped bacteria with lateral cell wall synthesis, DiIC12 produces a characteristic spotty pattern in exponential growth phase, marking regions of increased fluidity (RIFs), which harbor the lateral cell wall synthesis machinery (3, 17, 41, 54). DiIC12 is the only tool currently capable of detecting natural RIFs, apart from using RIF-associated proteins like MurG or PlsX as proxy (3).

RIFs have been shown to accumulate upon membrane depolarization (41), binding of antibiotics to lipid II (3, 4), and lipid phase separation (17). Since the fluidity of these domains may be altered (3), they are no longer referred to as RIFs but typically described as clusters or domains. As shown in Fig. 4B, all three antibiotics caused clustering of RIFs. However, valinomycin-treated cells still possessed regular RIFs in addition to large clusters, while cells treated with vancomycin and nisin did not exhibit any native RIFs anymore, presenting a smooth rest membrane. This can be explained by their different mechanisms. Membrane depolarization disturbs the localization of MreB, which organizes the regular distribution of RIFs and binds to the cell membrane via a similar mechanism as MinD (41, 44). When MreB forms clusters due to membrane depolarization, it concomitantly clusters RIFs but does not diminish the remaining microdomains (41). In contrast, vancomycin and nisin cluster lipid II. Since RIFs are lipid II-enriched cell wall synthesis domains (3–5, 9, 55, 56), this will deplete the rest membrane of lipid II and consequently RIFs. In the case of vancomycin, clusters are unrelated to the membrane potential, while in the case of nisin, both effects are present, leading to clusters formed due to both lipid II clustering and depolarization-dependent delocalization of MreB. A third possible mechanism of DiIC12 clustering is direct phase separation as observed with the peptide cWFW (34).

In line with our current understanding of these processes, DiIC12 clusters were also observed in cells treated with gramicidin, CCCP, tunicamycin, D-cycloserine, and tetracycline, but not with any of the other tested compounds (Fig. S13). Gramicidin and CCCP elicited comparatively minor changes in the Laurdan spectroscopy, while their effects on DiIC12 were prominent (Fig. S13), illustrating that overall membrane fluidity and phase separation into fluid and rigid domains can but do not have to go hand in hand (17). Similarly, tetracycline, nitrofurantoin, and ciprofloxacin affected overall fluidity but did not affect RIFs. These observations illustrate that more than one assay is needed to describe the complex factor that is cell membrane fluidity but also demonstrate the difficulty that can arise with interpreting such results without expert knowledge of bacterial cell biology.

### Cell wall synthesis

Methods to detect cell wall synthesis inhibition are diverse, yet many of them rely on difficult-to-obtain, expensive, or unsafe reagents, e.g., purified cell wall components and fluorescently or radioactively labeled precursors. Fluorescent protein fusions are a cheap and easily accessible alternative that can be implemented in most labs. Different cell wall synthesis proteins have been shown to co-localize with RIFs, including MreB (17, 41, 54), the lipid I synthase MraY (17), and the lipid II synthase MurG (3). MurG shows a near-perfect co-localization with RIFs, and its localization is similarly growth phase dependent (17). While MreB localization correlates with RIFs and MraY displays a limited overlap with these membrane domains, neither of them co-localizes strictly with RIFs or displays the same growth phase dependency (17, 41). In contrast to MreB, MurG and MraY appear to be insensitive to dissipation of the membrane potential (3, 41). Thus, these three proteins can give an indication of whether cell wall synthesis is inhibited (3, 34).

**Fig 5:**
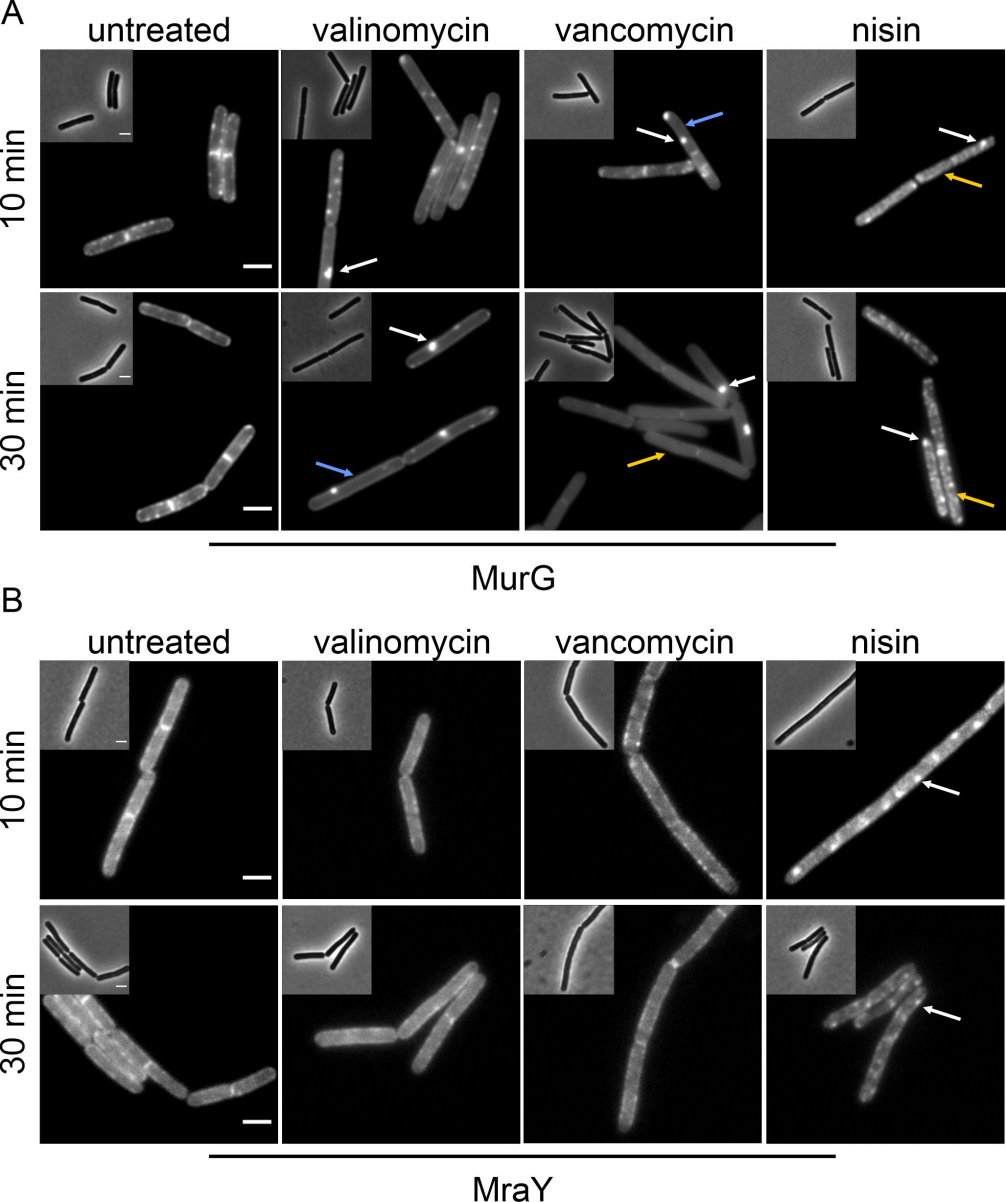
Effects of valinomycin, vancomycin, and nisin on cell wall synthesis proteins. (**A**) Localization of the lipid II synthase MurG [*B. subtilis* TNVS175 (*Pxyl-murG-msfgfp*)]. (**B**) Localization of the lipid I synthase MraY [*B. subtilis* TNVS284 (*Pxyl-mray-msfgfp*)]. White arrows indicate clustered protein. Blue arrows indicate cells with smooth membrane localization. Yellow arrows indicate cells with partially dispersed GFP signal. Scale bars 2 µm.

MurG was clustered into large foci by all three compounds. Yet, similarly to the DiIC12 stain, phenotypes differed with respect to non-clustered protein (Fig. 5A). Valinomycin caused clustering of MurG but did not abolish smaller foci, albeit less were visible after prolonged treatment. Vancomycin had a similar effect but entirely abolished small MurG clusters. In contrast, nisin displaced MurG into the cytosol. While these phenotypes reflect the compounds’ individual mechanisms, such differences can be hard to interpret by a non-expert.

The localization of MraY was not affected by valinomycin and vancomycin, while nisin caused clustering of the protein (Fig. 5B). Similar effects have been observed with other pore-forming peptides, suggesting that the displacement of MurG and MraY by nisin may be a consequence of large-scale membrane disruption (17).

**Fig 6:**
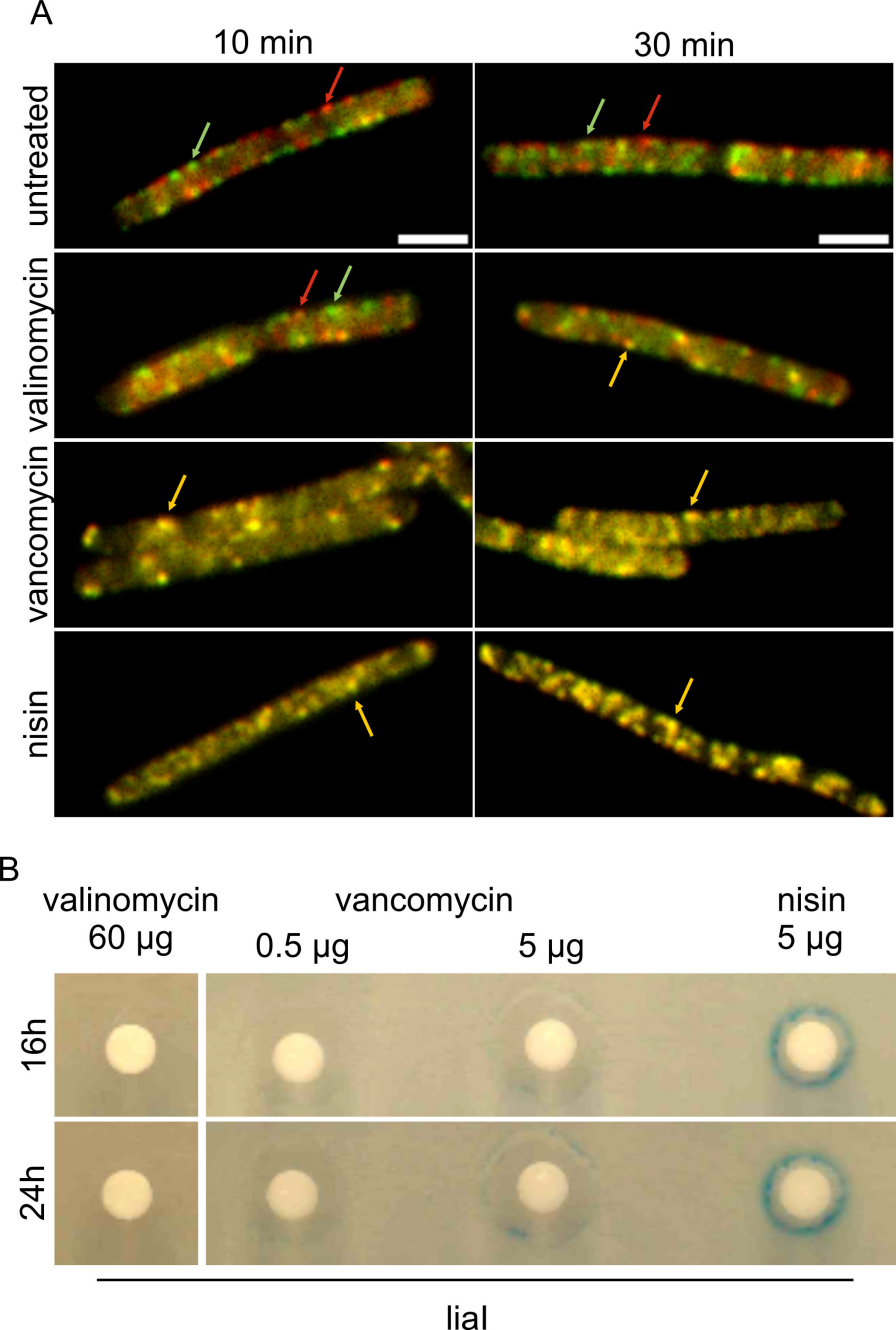
Effects of valinomycin, vancomycin, and nisin on MreB mobility and *PliaI* induction. (**A**) MreB mobility was assessed by recording two separate images of the same *B. subtilis* MW10 (*Pxyl-gfp-mreB*) cells in a 30-s interval. Individual images were false-colored red and green and overlaid, resulting in perfect overlap when MreB movement is stalled (yellow foci), and separate green and red foci when it is retained. Exemplary static foci are indicated by yellow arrows, while distinct red and green foci are indicated by arrows in the corresponding colors. Scale bar 2 µm. (**B**) Induction of *PliaI* in JB047 (*liaI-lacZ*) in a disk diffusion assay on agar plates containing X-gal. A blue ring around the inhibition zone indicates activation of the *PliaI* promoter.

MreB orchestrates lateral cell wall synthesis and is clustered by cell wall synthesis inhibitors, but it is also sensitive to dissipation of the membrane potential, reacting with clustering or loss of membrane binding (3, 41, 44). This makes its localization an unreliable readout for cell wall synthesis inhibition. However, MreB moves in a spiraling movement along the long axis of the cell, driving forward cell wall synthesis and ensuring rod shape (57–59). This movement is dependent on lipid-linked cell wall precursors, and all cell wall synthesis inhibitors tested so far have stalled MreB movement, while even aggressive membrane-active compounds did not completely abolish MreB mobility (60–63). Thus, MreB mobility can be used as a specific and robust reporter for cell wall synthesis activity. While time-lapse microscopy of MreB can be challenging due to the effects of oxygen supply and temperature on membrane potential and fluidity, MreB mobility can easily be assessed by simply taking two consecutive images of the same field of view in a 30-s interval. When overlaid, the two images will show a perfect overlap, if MreB is static, while distinct foci will be visible otherwise. Indeed, Fig. 6A shows normal MreB mobility in a cell treated with valinomycin, while vancomycin and nisin both arrested its movement. Stalled MreB movement was also observed with tunicamycin and D-cycloserine (Fig. S14) as well as with all previously tested cell wall synthesis inhibitors (61–63). Membrane-active compounds may detach MreB from the cell membrane (see gramicidin and CCCP; Fig. S14) or induce distinct, static clusters, yet as long as non-clustered MreB remains at the cell membrane, it retains its mobility (61–63). None of the other test compounds affected MreB mobility either (Fig. S14), making it a specific and robust readout for cell wall synthesis inhibition superior to localization of MurG and MraY.

While GFP fusions can be very useful for mode of action analysis, they are not always functional. Also, all membrane proteins are sensitive to large-scale membrane phase separation to some degree, making them less suitable as reporters for certain classes of antibiotics (17, 34). It can therefore be useful to complement microscopic experiments with genetic reporters. The two-component system LiaRS is known to respond to cell wall synthesis inhibition on lipid II level (32, 64). Hence, promoter activity of *PliaI* can be used as reporter for cell wall stress (38). Interestingly, the *PliaI* promoter specifically reacts to inhibition of a membrane-bound step of cell wall synthesis and is not activated by, e.g., β-lactam antibiotics or D-cycloserine, which inhibit cell wall synthesis at extracellular and intracellular steps, respectively (39, 65). It is also more strongly activated when the antibiotic binds in close proximity to the cell membrane yet does not require membrane damage for induction (32).

Here, we employed a disk diffusion assay using a strain that carries a LacZ reporter under control of *PliaI*. On X-gal-containing plates, promoter activation results in a blue halo around the inhibition zone (38, 64). As expected, valinomycin did not induce *PliaI,* while nisin caused a strong induction. Vancomycin showed a small effect, which is in line with its binding site at the D-ala-D-ala group, which is far removed from the lipid II membrane anchor (32, 64, 65). We also observed an induction with tunicamycin, which binds to MraY and inhibits lipid I synthesis, but not with any of the other test compounds (Fig. S15).

While it is not a universal reporter for cell wall synthesis inhibition, *PliaI* allows identification of inhibitors that bind to lipid-linked cell wall precursors including bactoprenol phosphate and pyrophosphate (32). The promoter may also react to indirect inhibition of the lipid II cycle, e.g., by clustering of RIFs or dissociation of MurG (24, 34). Therefore, it can be a valuable complementation of the MreB mobility assay, which reacts to all types of direct cell wall synthesis inhibition but not to membrane-mediated, indirect effects on this pathway (61–63). Together, these two assays allow reliable identification of cell wall synthesis inhibitors as well as narrowing down of the target to the membrane-bound lipid II cycle as opposed to intracellular and extracellular steps of cell wall synthesis.

### Conclusion

**Fig 7:**
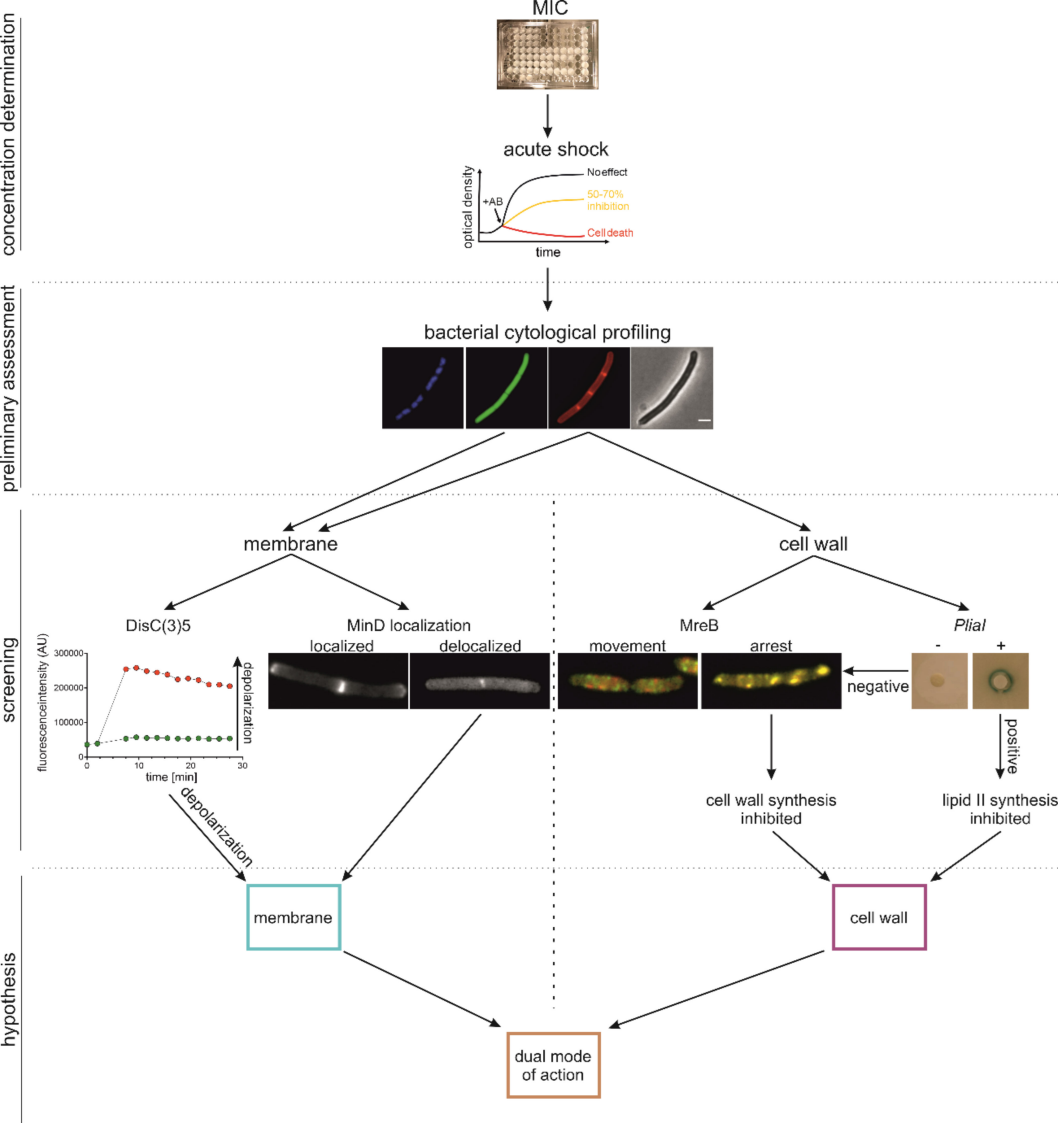
Flowchart of the suggested minimal phenotypic analysis assay kit for cell envelope-targeting antimicrobials. In a first step, the optimal stressor concentration of the test compounds is determined by minimal inhibitory concentration (MIC) and acute shock assays, followed by a first assessment of the mode of action by BCP, giving insight into overall cell (phase contrast), membrane (Nile red), and DNA (DAPI) morphology as well as intracellular content leakage (GFP). In a third step, mode of action assays branch into membrane [DiSC_3_(5) and MinD-GFP] and cell wall assays (MreB mobility and *PliaI* activation). These assays suffice to categorize a compound as membrane (blue box), cell wall (violet box), or dual-action inhibitor (brown box) as well as to exclude the cell envelope as a target, and thus aide steering follow-up assays for a more detailed mode of action analysis.

Based on this work and our experience with other BCP and phenotypic assays, we propose a minimal set of assays to distinguish between membrane and cell wall-active compounds and identify dual-action inhibitors, consisting of BCP, a suitable membrane potential assay, and MreB mobility (Fig. 7). If indicated, we suggest complementing these assays with Laurdan-based fluidity measurements and the *PliaI* reporter. BCP will give a first indication on whether the compound under investigation targets the cell envelope at all (Nile red) and rule out the formation of large pores (GFP). A membrane potential assay will then identify compounds that target the cell membrane. Thereby, we suggest that a dye-based assay be combined with a fluorescent protein fusion reporter to balance out their respective weaknesses. MreB mobility allows reliable identification of cell wall synthesis inhibitors. Complementing this assay with *PliaI* induction further allows narrowing down the target to the lipid II cycle. While relatively rare, some membrane-active compounds do not act by depolarization (3, 34). Thus, in cases where both depolarization and MreB mobility assays turn out negative, but BCP shows membrane defects, we suggest following up with a Laurdan spectroscopy assay if a spectrometer with suitable filters or monochromator is available. This set of assays is based on commercially available and affordable fluorescence dyes and easily available *B. subtilis* strains that can be grown and handled in most labs. Assays can be performed on fluorescence spectrometers and wide-field microscopes with standard filters (with the exception of the optional Laurdan assay) and detailed experimental protocols are available (2, 21, 48, 49) (see also Text S3 to S7). We believe that this set of assays can serve as a cheap and robust “mode of action starter kit” for cell envelope-targeting compounds that can be implemented, executed, and interpreted even by non-expert researchers. Further, it provides a good starting point for more detailed mode of action analysis and can therefore also serve as a basic assay set in more experienced labs.

## MATERIALS AND METHODS

### Strain and growth conditions

Strains used in this study are listed in Table S4. Unless otherwise noted, all strains were aerobically grown in Mueller-Hinton broth (MHB, Merck) at 30°C. Valinomycin requires the presence of high-potassium concentrations to exert its action (21). Thus, cells treated with valinomycin were grown in MHB containing 300-mM KCl (KCl-MHB). Untreated controls were always examined in both MHB and KCl-MHB (KCl-MHB controls are only shown for assays where the medium made a difference for the phenotype of the untreated control). Media were supplemented with the appropriate concentrations of xylose as specified in Table S4. For comparative purposes, selected experiments were additionally performed in LB, LB containing 300-mM KCl instead of NaCl, Belitzky minimal medium (66), and Spizizen minimal medium (67), or at 37°C. Unless otherwise noted, all assays were performed in biological triplicates.

### MICs

MICs were performed according to a modified version of the broth microdilution assay recommended by the Clinical Laboratory Standardization Institute. In short, twofold serial dilutions of antibiotics were prepared in MHB or LB in a 96-well microtiter plate. *B. subtilis* 168CA was added to a final colony-forming unit (CFU) count of 5 × 10^5^ CFU/mL from an exponential growing culture. Cells were incubated for 16 h at 30°C for MHB and 37°C for LB. In the case of valinomycin, cells were incubated in media containing 300-mM KCl. The MIC was determined as the lowest concentration inhibiting visible growth.

### Acute shock growth experiments

Growth experiments were performed by measuring the optical density (OD) of cells grown under constant agitation with the spectrophotometer GENESYS 30 (Thermo Scientific). Ten milliliters of *B. subtilis* 168CA was grown in MHB 30°C or LB 37°C until an OD_600_ of 0.3 was obtained. Cells were then split into 2-mL aliquots and treated with antibiotics to determine the optimal stressor concentration. In the case of valinomycin, cells were grown in media containing 300-mM KCl.

### Fluorescence light microscopy

All microscopy images were acquired on a Nikon Eclipse Ti2 equipped with a CFI Plan Apochromat DM Lambda 100X Oil objective (N.A. 1.45, W.D. 0.13 mm), a Photometrics PRIME BSI camera, a Lumencor Sola SE II FISH 365 light source, and an Okolab temperature incubation chamber. Images were obtained using the NIS elements AR software version 5.21.03 and analyzed with ImageJ (68).

### Bacterial cytological profiling

Bacterial cytological profiling was performed as described by Wenzel et al. (17). *B. subtilis* 168CA was grown to an OD_600_ of 0.3 and subsequently treated with antibiotics. Samples were taken after 10 and 30 min of antibiotic treatment. Cells were stained with 0.5-µg/mL Nile red (Invitrogen) and 1-µg/mL DAPI (Invitrogen) for 5 min. The samples were then spotted on 1.2% agarose films and imaged immediately. Images were processed with ImageJ (68).

### Protein localization experiments

All protein fusion strains except TB35 (*Pxyl-gfp-minD*), were grown overnight in medium supplemented with their respective inducer concentrations, diluted in the same medium on the next day, and grown to an OD_600_ of 0.3 prior to antibiotic treatment. Samples were taken for microscopy after 10 and 30 min. TB35 was handled correspondingly except that xylose was added only at the dilution step and not overnight.

### Image analysis

GFP intensity, membrane stress, and nucleoid compaction were analyzed in MicrobeJ (69). For quantification of GFP signal intensity, the parameters for bacterial recognition were set for medial-axis detection with an area of 0.5 max (μm). All other parameters remained at default settings. Cells were separated in accordance with the membrane stain. Out of focus cells as well as cells that were lysed were excluded from the analysis. For membrane stress quantification, the detected cells were manually counted according to the categories “stressed” (bright foci in the membrane stain) and “normal” (smooth membrane stain). For DNA compaction analysis, the maxima of foci detection were used. The parameters within the maxima detection remained at default settings. The Z-score and tolerance were adjusted manually to ensure fitting DNA detection. The compaction was calculated based on the quotient of the cell area divided by the DNA area.

### DiSC_3_(5) spectroscopy

DiSC_3_(5) stock solutions were prepared at 100 µM in sterile DMSO and stored at −20°C. Measurements were performed according to Winkel et al. (21) with minor modifications. *B. subtilis* 168CA was grown in the presence of 50 µg/mL Bovine albumin serum (BSA). After reaching exponential growth phase (OD_600_ 0.3), 1 µM DiSC_3_(5) was added to the cells. A dimethyl sulfoxide (DMSO) concentration of 1% was constantly maintained to prevent precipitation of the dye. Antibiotics were added after the fluorescence baseline had stabilized. Measurements were run for 30 min after antibiotic addition. The assay was performed in 96-well black polystyrene microplates (Corning) using a BMG Clariostar Plus plate reader at an excitation wavelength of 610 nm with a bandwidth of 30 nm and an emission wavelength of 675 nm with a bandwidth of 50 nm.

### Laurdan spectroscopy

Kinetic membrane fluidity measurements were performed as described previously (48) with minor modifications. *B. subtilis* 168CA was grown in medium containing 0.2% glucose. After reaching an OD_600_ of 0.6, cells were stained with 10-µM Laurdan (AnaSpec) for 5 min, washed in Laurdan buffer [phosphate-buffered saline, 0.2% glucose, 1% dimethylformamide (DMF)], and resuspended in the same buffer to an OD_600_ of 0.8. A DMF concentration of 1% was constantly maintained to prevent precipitation of the dye. Sample aliquots of 100 µL were added to 96-well black polystyrene microplates (Corning), and fluorescence was measured at an excitation wavelength of 350 nm and emission wavelengths of 460 nm and 500 nm with 15 nm bandwidth each. After recording the baseline for 5 min, 100 µL of prewarmed Laurdan buffer containing the respective antibiotics was added and measurements were continued for another 30 min. Cells grown in KCl-MHB were washed and resuspended in Laurdan buffer containing 300-mM KCl. General polarization values of Laurdan were calculated according to Wenzel et al. (48).

### DiIC12

DiIC12 (AnaSpec) staining was performed as described in Saeloh et al. (16). In short, overnight cultures were diluted 1:200 in their respective media and stained with 2-µg/mL DiIC12. A DMSO concentration of 1% was constantly maintained to prevent precipitation of the dye. Stained cells were grown to an OD_600_ of 0.3 and washed four times with prewarmed medium containing 1% DMSO. After resuspension, the culture was split and treated with antibiotics for 10 and 30 min, respectively.

### X-gal disk diffusion assay

*B. subtilis* JB048 (*liaI-lacZ*) (64) was grown to an OD_600_ of 0.3 prior to plating on agar plates containing 100-µg/mL X-gal (Fisher Scientific). Antibiotic-containing filter paper disks were placed on the plates, and samples were incubated at 30°C for 16 h. Since the activity of antibiotics can vary between liquid and solid media, concentrations were chosen that resulted in a ~2-cm inhibition zone: 60.0-µg valinomycin, 5.0-µg nisin, and 0.5- and 5.0-µg vancomycin.

## SUPPLEMENTAL MATERIAL

Supplementary material spectrum.03275-23-s0001.pdf Supplementary text, tables, and figures.

ASM does not own the copyrights to Supplemental Material that may be linked to, or accessed through, an article. The authors have granted ASM a non-exclusive, world-wide license to publish the Supplemental Material files. Please contact the corresponding author directly for reuse.
