# Supplementary material for: Dissecting antibiotic effects on the cell envelope using bacterial cytological profiling: a phenotypic analysis starter kit
Source: Microbiol Spectr. 2024 Jan 30;12(3):e03275-23. doi: 10.1128/spectrum.03275-23 (PMC10913488; doi:10.1128/spectrum.03275-23)
Supplement: Supplementary material — Supplementary text, tables, and figures. [file spectrum.03275-23-s0001.pdf]

Supplementary Material

Dissecting antibiotic effects on the cell envelope using bacterial cytological profiling: A phenotypic analysis starter kit

Ann-Britt Schäfer[1,2], Margareth Sidarta[1,2], Ireny Abdelmesseh Nekhala[1], Gabriela Marinho Righetto[1,2], Aysha Arshad[1], Michaela Wenzel[1,2]*

[1]Division of Chemical Biology, Department of Life Sciences, Chalmers University of Technology, 412 96 Gothenburg, Sweden

[2]Center for Antibiotic Resistance Research in Gothenburg (CARe), Gothenburg, Sweden

*Corresponding author: wenzelm@chalmers.se

Contents

TEXT S1 Selecting antibiotic concentration, growth conditions, and fluorescence dyes for phenotypic analysis.

TEXT S2 Quantitative image analysis of microscopic phenotypic analysis assays.

TEXTS3 Step-by-step protocol for Nile red, DAPI, and GFP microscopy.

TEXT S4 Step-by-step protocol for DiSC(3)5 spectroscopy.

TEXT S5 Step-by-step protocol for laurdan spectroscopy.

TEXT S6 Step-by-step protocol for DiIC12 staining.

TEXT S7 Step-by-step protocol for *PliaI* disc diffusion assay.

TABLE S1 Minimal inhibitory and optimal stressor concentrations for valinomycin, vancomycin, and nisin.

TABLE S2 Optimal stressor concentrations and reported mechanisms of action of comparator compounds.

TABLE S3 Summary of effects of comparator compounds in each assay.

TABLE S4 Strains used in this study.

FIG S1 Growth inhibition of *B. subtilis* 168CA by valinomycin, vancomycin, and nisin in MHB at 30 °C.

FIG S2 Growth inhibition of *B. subtilis* 168CA by valinomycin, vancomycin, and nisin in LB at 37 °C.

FIG S3 Media compatibility of Nile red and mitotracker green. All cells were grown at 30 °C and stained in mid-exponential growth phase.

FIG S4 Selection of membrane dye concentration.

FIG S5 Influence of growth phase, temperature, and medium on DiIC12 staining of *B. subtilis* 168CA.

FIG S6 Influence of induction time on MinD localization.

FIG S7 Selective inner membrane staining of outer membrane-permeable *E. coli* cells.

FIG S8 Phase contrast images of healthy, phase-dense cells (left) and lysing cells losing phase contrast (right).

FIG S9 Nucleoid compaction analysis of *B. subtilis* MW54 (*PrpsD-msfGFP*) treated with valinomycin, vancomycin, and nisin.

FIG S10 Quantification of intracellular GFP intensity **(A)** and membrane damage **(B)** of *B. subtilis* MW54 (*PrpsD-msfGFP*) treated with valinomycin, vancomycin, and nisin.

FIG S11 Bacterial cytological profiling of *B. subtilis* bSS82 (*PrpsD*-*gfp*) treated with antibiotics for 10 and 30 min.

FIG S12 Effects of antibiotics on the membrane potential.

FIG S13 Effects of antibiotics on membrane fluidity.

FIG S14 Effects of antibiotics on MreB mobility.

FIG S15 Effects of antibiotics on *PliaI* induction.

TEXT S1 Selecting antibiotic concentration, growth conditions, and fluorescence dyes for phenotypic analysis.

Selection of antibiotic concentrations

Choosing the right antibiotic concentration and treatment time is crucial for successful phenotypic analysis. Too low concentrations will not lead to visible effects, while too high concentrations can kill or lyse the cells, leading to pleiotropic effects due to cell death and disintegration. Even for bacteriostatic antibiotics an overdose can lead to off-target activities that would not be observed at inhibitory concentrations. Likewise, treatment time can be detrimental. In order to observe the direct effects of an antibiotic, short treatment times are indicated. In our experience, 10 to 15 minutes tend to be suitable for most antibiotics. However, some compounds require longer treatment times to show an effect e.g., beta-lactams, fluoroquinolones, or cell division inhibitors, whose effects only become apparent after one or more rounds of cell division have (or should have) occurred (1).

Simply choosing the MIC is not sufficient in many cases, as the conditions differ between MIC incubation and phenotypic experiments with respect to cell density, growth phase, culture volume, and aeration, to name a few. Similarly, killing kinetics are not suitable to determine the optimal treatment time as dead cells should be avoided. Therefore, acute shock experiments to determine the growth behavior after antibiotic addition in log phase are the method of choice (see **Figure S1**). Usually, a concentration leading to a 50-70% reduction in growth rate is suitable for phenotypic experiments (referred to as optimal stressor concentration, OSC) (2). However, for some compound classes, in particular membrane-active molecules, this window can be hard to near-impossible to find as they tend to result in an 'all-or-nothing' response. In such a case, it can be necessary to choose multiple concentrations and monitor the growth rate during each experiment to ensure that growth inhibition but no lysis occurred (3). Some compounds have a two-staged mechanism of action e.g., nisin which binds to lipid II and after reaching a critical concentration forms a transmembrane pore. In this case, in order to capture both activities of the compound, slightly higher, near-inhibitory concentrations have to be used. Accordingly, in this study we used near-inhibitory concentrations of all antibiotics to have comparable stress levels under the different conditions (**Figure S1**).

Selection of growth conditions

It is pivotal to perform acute shock experiments under the same conditions phenotypic analysis is carried out. Likewise, conditions should be the same for all phenotypic assays in a study, as far as the specific experimental setups allow. Like antibiotic concentration and treatment time, media composition, growth temperature, culture volume, and aeration, to name a few, can influence how cells grow, how they present phenotypically, and how well an antibiotic compound works.

Media composition is a crucial and well-known factor that influences antibiotic activity (4, 5). Even for very similar media like MHB and LB, which are both full media commonly used for microbiology, significant differences can be found in MIC (e.g., 8-fold for nisin, **Table S1**). Likewise, the OSCs differ considerably (e.g., 5-fold for valinomycin, **Table S1**, **Figure S1-2**). Thus, conditions must be kept as similar as possible and if an antibiotic requires a specific supplement (e.g., valinomycin requiring KCl media), a separate untreated control culture in the modified medium is essential.

Selection and compatibility of dyes

Fluorescence dyes are crucial for BCP and many other phenotypic assays, but not all dyes are suitable for all setups e.g., many membrane dyes display phototoxicity and are therefore not suitable for timelapse microscopy (6). Importantly, some membrane dyes are not compatible with certain culture media. Thus, both Nile red and mitotracker green deliver good membrane stains in full media like MHB and LB. Yet, mitotracker green leads to pronounced membrane stress in minimal media such as BMM and SMM (**Figure S3**), making it unusable under these conditions. Similarly, dye concentration is crucial. For example, too low concentrations of Nile red and mitotracker green lead to unclear and blurry membrane stains while too high concentrations display toxicity that becomes apparent as clear membrane stress phenotypes. Likewise, an overdose of DAPI leads to membrane stress and cell lysis (**Figure S4**). Staining times can be crucial as well. Too short incubation leads to heterogenous stains while too long incubation may lead to phenotypic effects due to toxicity. For BCP dyes, 5 min has been proven adequate in our hands.

DiIC12, which stains fluid membrane domains is strongly affected by a number of conditions as RIFs behave differently at different growth phases, temperatures, and media conditions (**Figure S5**). We have made similar observations for RIF-associated proteins like MurG and PlsX (data not shown).

When working with fluorescent protein fusions, inducer concentrations and induction times can be critical as well. For example, we typically grow strains expressing GFP fusions to MurG, MraY, and MreB in constant presence of inducer. However, TB35 expressing

GFP-MinD requires shorter induction times and lower inducer concentrations as overexpression of MinD leads to inhibition of cell division. Hence, elongated cells with disturbed localization patterns are observed (**Figure S6**).

Selection of model organism

In this study, which was aimed at evaluating assays that are accessible for most labs/researchers, we have chosen *B. subtilis* as model because it is non-pathogenic and can be grown in most labs. It is sensitive to most antibiotics and has been used extensively for antibiotic mode of action studies in the past, including phenotypic analysis studies, making it a very good reference organism. Further, the availability of a range of different strains and libraries offers near-endless possibilities for deeper cell biological characterization of antibiotic mechanisms. However, it is of course possible to employ BCP and other phenotypic assays to other species including pathogens. Thus, adaptation to other Gram-positive bacteria is usually seamless (7, 8).

Gram-negative bacteria, such as *E. coli* can pose more of a challenge as their outer membrane does not permit the passage of many dyes. However, outer membrane-permeable strains e.g., overexpressing outer membrane porins (9, 10), or selective outer membrane-permeabilizing agents e.g., polymyxin B nonapeptide (11), can solve this issue. Yet, for BCP specifically another problem is the specificity of membrane dyes as the vast majority of dyes stain both the inner and outer membrane, even when the outer membrane is permeabilized. However, mitotracker green appears to be inner membrane-selective, yet outer membrane-impermeable, enabling specific inner membrane visualization in outer membrane-permeabilized cells (**Figure S7**). An alternative strategy to visualize membrane defects is using an ubiquitous membrane protein fused to GFP e.g., GlpT (1, 12–14), as proxy.

TEXT S2 Quantitative image analysis of microscopic phenotypic analysis assays.

While the experienced eye can easily interpret microscopy images from appearance alone, quantification can aid interpretation by less experienced researchers, increase comparability between labs, and visualize effects of population heterogeneity. Licensed microscope software such as NIS Elements (Nikon) or Zen (Zeiss) have inbuilt functions for image analysis that are suitable for most basic needs. However, freely available software is more popular as it allows easy transfer of image analysis workflows between labs without the obstacle of licensing and operating different software interfaces. ImageJ is typically the program of choice, mostly due to its plethora of functions and easy customization through plugins and macros. Programs based on ImageJ that are specifically tailored to the needs of analyzing bacterial cells are ObjectJ (developed for *Escherichia coli* and later amended with plugins aiding single-cell analysis of *B. subtilis* cells growing in chains) (15–17) and MicrobeJ (developed for use on various microbial cells) (18).

These tools enable automated detection and quantification of bacterial cells based on phase contrast or fluorescence intensity. Details on these programs and their capacities can be found in the aforementioned references. Here, we want to briefly mention workflows for quantification of the phenotypic analysis assays used in this study and address typical limitations and pitfalls of automated or semi-automated image analysis.

One major challenge when working with *B. subtilis* is that it forms a cell division septum before cells separate, resulting in chains of cells that are indistinguishable by phase contrast. This poses a challenge for programs like ObjectJ that are optimized for *E. coli*, in which septation and separation occur simultaneously, allowing easy single cell detection by phase contrast alone (17). One solution to this problem are the plugins Chain Tracer and Nuc Tracer, which were developed to automatically detect single cells based on membrane and nucleoid stains, respectively (17). An alternative is MicrobeJ, which can also detect cells based on membrane stains but also allows manual separation of cells (18). Once single cell detection is established, quantitative analysis of phenotypes is feasible.

Quantification of BCP

Since BCP is a combination of methods, several image analysis workflows must be employed to quantify each parameter. Phase contrast can deliver information on cell morphology. MicrobeJ can easily detect cell length and width, parameters that are of importance for cell division inhibitors and certain antibiotics targeting cell wall synthesis (e.g., fosfomycin, which leads to shape deformations).

Likewise, the program can automatically quantify fluorescence intensity per cell, which can be used to quantify GFP (or dye) leakage and identify pore-forming compounds (**Figure S10**). While the image analysis is straight-forward in this case, pore assays themselves are sensitive to the conditions and need to be carefully controlled. Thus, cell lysis will lead to the leakage of intracellular content as well as the uptake of pore dyes such as propidium iodide and Sytox Green, leading to false-positive results, especially in species like *B. subtilis*, which is prone to undergoing autolysis under unfavorable conditions. Therefore, careful selection of antibiotic concentrations and treatment times as well as lysis controls are paramount. Since phase contrast can serve as internal control for cell lysis, it is possible to exclude single lysed cells from the analysis or include them and state their abundance. This is not possible when using spectroscopic batch measurements or even flow cytometry and is a clear advantage of microscopic assessment of pore formation.

Nucleoid compaction can be quantified in a manner similar to whole-cell fluorescence. To this end, the DAPI signal is detected and its area compared to the area of the whole cell based on phase contrast (**Figure 9A**). One limitation of this analysis is the fluorescence intensity. Some compounds diminish or eliminate the DAPI stain e.g., nitrofurantoin and peroxide, which at higher concentrations lead to DNA disintegration (9, 19). In such cases, the analysis will not result in sensical data. For compounds with such clear phenotypes, this is not a problem in praxis as the effects are usually abundantly clear without the need to quantify. However, in cases with pronounced population heterogeneity, this can be a major problem. This was the case for valinomycin in this study. While nucleoid relaxation was clearly visible by eye (**Figure 2**), image analysis showed no clear difference in nucleoid compaction values (**Figure 9A**). This is due to the reduced or absent DAPI signal in the portion of cells that show nucleoid relaxation (**Figure 9B**). While this phenotype makes sense in light of the proposed mechanism of DNA fragmentation (20), it will be excluded from DNA compaction analysis as the software will not detect the fluorescence signal. Such examples illustrate that, while image analysis can be very useful, it must always be double-checked by manual assessment and compared to the phenotypes observed by eye.

Membrane foci in the Nile red (or any other membrane) stain can in principle be analyzed by foci detection. However, in practice, we have made the experience that it is near-impossible to accurately capture all foci due to the variety in foci size and fluorescence intensity as well as the inability of the software to distinguish an aberrant membrane focus from a freshly forming cell division septum. While this limitation could be overcome in the future e.g., with the implementation of machine learning plugins, the currently most reliable quantification

method is simple counting of phenotypes. This can be facilitated by the cell straightening function in MicrobeJ, which displays all detected cells in an organized and numbered manner. Thus, while the analysis does rely on visual inspection and is therefore not unbiased, the datasets can easily be made available for independent assessment and reevaluation.

Quantification of GFP localization

In principle, GFP localization can be quantified similarly to BCP. However, the method must be adapted for each fusion depending on the localization pattern. Thus, intracellular proteins can be quantified in intensity in the same manner as GFP and nucleoid-associated proteins may be detected analogously to nucleoid compaction. Longitudinal line scans, either manual in ImageJ or automated in ObjectJ, are suitable for most cell division proteins that localize at mid-cell, cell poles, or both (21) e.g., MinD (**Figure 3**). Loss of membrane binding of evenly distributed membrane proteins, such as MraY, can be analyzed with line scans across the cell. Membrane proteins that localize in foci suffer from the same foci detection problems as membrane stains, making automated analysis difficult, and often require manual counting.

Quantification of MreB movement can be done in two ways, kymographs and co-localization analysis. Kymographs are static representations of a series of timelapse images and visualize movement. For labs equipped for extended live cell imaging, kymographs constitute a powerful visualization tool. Yet, the sensitivity of MreB to oxygen limitation (22) makes timelapse imaging difficult or impossible, when the required conditions are not met, and is thus not possible in every lab.

In principle, it is possible to quantify the co-localization between MreB foci from only two individual images as displayed in **Figure 6** and **S14**, using the co-localization function in ImageJ. However, it must be noted that the fast movement of MreB results in fully mobile foci overlapping by chance. Additionally, the propensity of MreB to partially lose its membrane binding under stress creates a general cellular fluorescence background, which may result in a false-positive analysis. Thus, simple overlay images or timelapse videos, looping the two individual images, often provide a clearer readout than co-localization analysis.

Conclusion

While many of the issues addressed here can in principle be solved by careful adjustment of image analysis parameters, in particular detection thresholding, the diversity of phenotypes elicited by antibiotics often makes it necessary to adjust parameters individually for each sample, sometimes for each image, or even for different cell populations within the same image, undermining the purpose of unbiased analysis and requiring rather extensive knowledge and experience in image analysis. To avoid analysis artefacts, researchers should always

manually check their automated analyses, investigate suspicious values, and carefully cross-evaluate their quantitative data with visual inspection.

TEXTS3 Step-by-step protocol for Nile red, DAPI, and GFP microscopy.

Day 1

- inoculate the respective strain in 2 mL MHB in a 50 mL culture tube and grow overnight at 30 °C[1].
    - if a GFP fusion strain is used, the medium is supplemented with appropriate concentrations of inducer (see **Table S4**).

Day 2

- 1:100 dilute the overnight culture in 2 mL fresh medium supplemented with appropriate inducer concentrations, where applicable, and grow at 30°C.
- allow cultures to grow to an OD_{600} of 0.3.
- transfer 100 µL of culture to 2 mL microtubes in a pre-warmed thermoshaker[2].
- add antibiotics of interest to the samples and incubate for the desired time (usually, short treatment times of 5-10 min give best results)
- if fluorescence staining is desired, add 0.5 µg/mL Nile red and 1 µg/mL DAPI 5 min prior to imaging[3].
- spot 0.5 µL of each sample on agarose-coated (1.2%) microscopy slides[4].

Notes:

[1]Media and growth temperatures may be adjusted as necessary. *B. subtilis* is sensitive to oxygen availability and the ratio of liquid to tube/flask volume is critical. Furthermore, culturing *B. subtilis* in reusable glassware may result in pre-stressed cells due to remnants of cleaning agents. In our hands, 2 mL medium in 50 mL single-use plastic culture tubes have proven optimal for small-scale experiments.

[2]Maintenance of constant temperature and shaking is crucial as both temperature shifts and oxygen depletion elicit effects similar to membrane-acting compounds and drastically affect phenotypic analyses.

[3]Both dyes are toxic to bacterial cells after extended exposure, hence longer staining must be avoided. Shorter staining may result in sub-optimal images. Higher dye concentrations are toxic even at short exposure and lead to drastic phenotypic effects.

[4]Please refer to te Winkel *et al.* (23) for slide preparation.

TEXT S4 Step-by-step protocol for $DiSC_35$ spectroscopy.

Detailed descriptions and protocol variations including alternative dyes are described in detail in te Winkel *et al*. (23) and Buttress *et al*. (11).

Day1:

- inoculate the respective strain (here *B. subtilis* 168CA) in 2 mL MHB supplemented with 50 µg/mL BSA[1] in a 50 mL culture tube and grow overnight at 30 °C[2].

Day2:

- 1:100 dilute the overnight culture in 2 mL fresh medium supplemented with 50 µg/mL BSA and grow at 30 °C.
- while the cells are growing, prepare the following solutions:
    - dilute 100 µM $DiSC_35$ stock solution to 15 µM $DiSC_35$ working solution in MHB containing 50 µg/mL BSA.
    - dilute antibiotics[3] to 100x of the test concentration in a minimum volume of 10 µL. Gramicidin can be used as a positive control at a concentration of 1 µg/mL.
- when the culture reaches an OD_{600} of 0.3[4], add 138.5 µL to the wells of a black polystyrene microtiter plate.
- transfer the plate to a suitable fluorescence platereader[5] and start the measurement: excitation wavelength 610 +/-30 nm, emission wavelength 675 +/-50 nm, minimum intervals.
- after three measurements (medium-only baseline), pause the run and add 10 µL of 15 µM $DiSC_35$ working solution to each well and restart the run.
- continue the measurements until the fluorescence baseline is stable.
- pause the run and add 1.5 µL of 100x concentrated antibiotics to each sample.
- restart the run and continue readings for 30 minutes[6].

Notes:

[1]BSA helps keeping the baseline stable in the spectroscopic measurements. In order to avoid any effects of media changes, it is added to the cultures from the start of the experiment.

[2]Media and growth temperatures may be adjusted as necessary.

[3]When testing a new antibiotic, interference with $DiSC_35$ should be tested by measuring the fluorescence of the dye in the absence of cells. To this end, follow the same protocol but add sterile medium containing 50 µg/mL BSA to the wells instead of bacterial culture.

[4]Exponentially growing cultures can be diluted down to an OD_{600} of 0.3, but it should be avoided diluting from transition/stationary phase cultures as those display different membrane potential levels and higher population heterogeneity, leading to less reproducible results.

[5]For the same reasons mentioned in the previous protocol, the instrument should be temperature-controlled and capable of continuous shaking.

[6]Longer measurements are affected by photobleaching and endpoint measurements may be a better choice. Please refer to Schäfer *et al*. (9) for experimental procedures.

TEXT S5 Step-by-step protocol for Laurdan spectroscopy.

Detailed and varied protocols for Laurdan measurements including alternative dyes are described in detail in Wenzel *et al*., Scheinpflug *et al*., and Humphrey *et al*. (8, 24, 25).

This assay is particularly temperature-sensitive. Temperature shifts must be avoided. All consumables, including tubes, microplates, pipette tips, etc., as well as media and buffers must be pre-warmed to the desired temperature (here, 30 °C).

Day1:

- inoculate the respective strain (here, *B. subtilis* 168CA) in 2 mL MHB supplemented with 0.2% glucose[1] in a 50 mL culture tube and grow overnight at 30 °C[2].

Day 2:

- 1:100 dilute the overnight culture in 2 mL fresh medium supplemented with 0.2% glucose and grow at 30 °C.
- while the cultures are growing, prepare the following:
    - dilute Laurdan stock solution (Sigma 40227, 10 mM in 100% DMF) to a working solution of 1 mM Laurdan in 100% DMF.
    - prepare Laurdan buffer (PBS, 1%DMF, 0.2% glucose) and pre-warm to 30 °C.
    - dilute antibiotic stock solutions to 100x of the final concentration in a minimum volume of 20 µL. Benzyl alcohol can be used as a positive control at a concentration of 100 mM.
    - prepare microtiter plate by adding 147 µL of Laurdan buffer and 3 µL of the respective antibiotic solutions[3]. Keep the plate at 30 °C until the measurement starts.
- after cultures reach an OD_{600} of 0.6, add 10 µM Laurdan (from 1 mM stock) and incubate for 5 min.
- wash cells four times in pre-warmed Laurdan buffer using short centrifugation steps of 30 s at 16 000 × g.
- after the last washing step, adjust the OD_{600} to OD 0.8.
- add 100 µL of sample per well of a black polystyrene microplate and measure fluorescence baseline: excitation wavelength 350 +/-15 nm, emission wavelengths 460 +/-15 and 500 +/-15-nm, 2 min intervals.
- after three measurements, pause the run and add 100 µL of the prepared buffer antibiotic mix to the samples.

- restart the run and continue readings for 30 min.
- calculate Laurdan general polarization using the formula GP = $(I_{460}-I_{500})/(I_{460}+I_{500})$ and plot against time.

Notes:

[1]Measurements are conducted in buffer due to the autofluorescence of yellow-tinted culture media such as MHB. Glucose is added to maintain cell energization and membrane potential during washing and measurements. To avoid additional effects of media changes, glucose is added to the cultures from the start of the experiment. If experiments are conducted in colorless medium, measurements can be conducted in the same medium (i.e., washing and resuspending in medium instead of Laurdan buffer) and the addition of glucose can be omitted.

[2]Media and growth temperatures may be adjusted as necessary.

[3]When testing a new antibiotic, interference with Laurdan should be tested by measuring the fluorescence of the dye in the absence of cells. To this end, follow the same protocol but add sterile Laurdan buffer containing 10 µM Laurdan to the plate instead of the stained and washed bacteria.

TEXT S6 Step-by-step protocol for DiIC12 staining.

Detailed protocols for DiIC12 measurements are described in detail in Wenzel *et al*. and Humphrey *et al*. (8, 24).

Day1:

- inoculate the respective strain (here, *B. subtilis* 168CA) in 2 mL MHB in a 50 mL culture tube and grow overnight at 30 °C[1,2].

Day2:

- 1:200 dilute the overnight culture in MHB containing 1 µg/mL DiIC12 (AnaSpec AS-84902, 100 µg/mL stock in 100% DMSO)[3].
- after reaching an OD_{600} of 0.3[4], wash cultures four times with pre-warmed MHB containing 1% DMSO using short centrifugation steps of 30 s at 16 000 × g.
- resuspend cell pellets in MHB containing 1% DMSO and re-adjust to an OD_{600} of 0.3.
- transfer 100 µL of culture to 2 mL microtubes in a pre-warmed thermoshaker[5].
- add antibiotics of interest to the samples and incubate for the desired time (usually, short treatment times of 5-10 min give best results[6])
- spot 0.5 µL of each sample on agarose-coated (1.2%) microscopy slides[7].

Notes:

[1]Media and growth temperatures may be adjusted as necessary.

[2]The OD_{600} of the overnight culture should not exceed 4 as overly stationary cultures may display long lag phases and slower growth, which affects the distribution of membrane microdomains resulting in aberrant or smooth patterns.

[3]Maintenance of a final DMSO concentration of 1% is crucial to keep the dye in solution.

[4]Natural RIFs are only visible in fast-growing exponential cultures (26).

[5]Maintenance of constant temperature and shaking is crucial as both temperature shifts and oxygen depletion elicit effects similar to membrane-acting compounds and drastically affect phenotypic analyses.

[6]If excessive dye precipitation is observed (precipitation increases with treatment time), additional washing steps may be included immediately prior to microscopy.

[7]Please refer to te Winkel *et al*. (23) for slide preparation.

TEXT S7 Step-by-step protocol for *PliaI* disk diffusion assay.

Antibiotic activity can vary drastically between agar and liquid medium assays. To ensure that an appropriate inhibition zone (approximately 2 cm) is reached, initial concentration tests should be conducted on MHB agar plates before proceeding to *PliaI* activation assays.

Day 1:

- prepare X-gal stock solution (Fisher Scientific, 100 µg/mL in DMSO).
- prepare MHB agar plates containing 100 µg/mL X-gal and store in the dark at room temperature[1].
- inoculate *B. subtilis* JB048 (*liaI-lacZ*) in MHB containing 2 µg/mL erythromycin and grow overnight at 30 °C[2].

Day2:

- 1:100 dilute the overnight culture in MHB without erythromycin and grow at 30 °C.
- while the cells are growing, prepare antibiotic dilutions so that a maximum of 10 µL have to be added to the disk to reach the desired final concentration. As a positive control, 5 µg nisin can be used.
- when the cells reach an OD_{600} of 0.3, plate 100 µL of culture per agar plate[3].
- add the appropriate amounts of antibiotic solution to sterile 6 mm filter paper disks.
- place disks on the agar plate and gently press down[4].
- incubate agar plates over night at 30 °C in the dark.

Day 3:

- after 16 h of incubation, inspect agar plates for blue color around the inhibitions zones and take images with an appropriate scanner or camera.

Notes:

[1]Plates should be freshly prepared on the day of or before the experiment is conducted.

[2]Media and growth temperatures may be adjusted as necessary.

[3]If necessary, adjust volume so that a confluent layer of bacteria is achieved after overnight incubation.

[4]Mind the expected diameter of the inhibition zone and place discs accordingly.

TABLE S1 Minimal inhibitory and optimal stressor concentrations for valinomycin, vancomycin, and nisin.

	antibiotic	minimal inhibitory concentration	optimal stressor concentration
MHB, 30 °C	valinomycin	0.75 µg/mL	10 µg/mL
	vancomycin	0.125 µg/mL	0.5 µg/mL
	nisin	3.2 µg/mL	0.8 µg/mL
LB, 37 °C	valinomycin	2 µg/mL	2 µg/mL
	vancomycin	0.25 µg/mL	0.25 µg/mL
	nisin	6.4 µg/mL	3.2 µg/mL

TABLE S2 Optimal stressor concentrations and reported mechanisms of action of comparator compounds.

compound	concentration	mechanism of action
gramicidin	1 µg/ml	Na^+/K^+ channel ionophore
CCCP	100 µM	H^+ carrier ionophore
tunicamycin	16 µg/ml	cell wall synthesis (lipid I synthase MraY)
D-cycloserine	30 µg/ml	cell wall synthesis (alanine racemase and D-ala-D-ala ligase)
ciprofloxacin	1 µg/ml	DNA gyrase and topoisomerase IV
rifampicin	0.08 µg/ml	RNA polymerase
tetracycline	2.5 µg/ml	protein synthesis (30S ribosome subunit), membrane (19)
kanamycin	2 µg/ml	protein synthesis (30S ribosome subunit)
chloramphenicol	1 µg/ml	protein synthesis (50S ribosome subunit)
nitrofurantoin	28 µg/ml	macromolecule damage through generation of reactive species

TABLE S3 Summary of effects of comparator compounds in each assay.

		**gramicidin**	**CCCP**	**tunicamycin**	**D-cycloserine**	**ciprofloxacin**	**rifampicin**	**tetracycline**	**kanamycin**	**chloramphenicol**	**nitrofurantoin**
BCP	DAPI	+	+	-	-	-[#]	+	+	-	-	+
	Nile red	+	+	+	+	-[#]	-	+	-	+	+
	GFP	-	-	-	-	-	-	-	-	-	-
membrane	MinD	+	+	-	-	-	-	+	-	-	+
	DiSC$_3$5	+	na	-	-	-	-	-	-	-	-
	laurdan	(+)	(+)	-	-	+	-	+	-	-	+
	DiIC12	+	+	+	+	-	-	+	-	-	-
cell wall	MurG	+*	+*	+[&]	+[&]	-	+[&]	+[¤]	-	-	+[¤]
	MreB localization	+	+	-	-	-	-	-	-	-	-
	MreB mobility	na	na	+	+	-	-	-	-	-	-
	PliaI	-	-	+	-	-	-	-	-	-	-

[#]Clear effects on both DNA and membrane are observed after 1 h of treatment (1, 12, 13).
*Loss of membrane binding.
[&]Cluster formation.
[¤]Smooth localization, may be observed when growth is slowed or halted (1, 12, 13).
(+): Small and/or transient effect.
na: Not applicable due to interference of the compound with the assay.

TABLE S4 Strains used in this study.

**strain**	**genotype**	**induction**	**reference**
168CA	*trpC2*	-	(27)
bSS82	*trpC2 amyE::spc PrpsD-gfp*	-	(28)
TB35	*trpC2 amyE::spc Pxyl-gfp-minD*	0.1% xylose	1981->168CA (29)
MW10	*trpC2 amyE::spc Pxyl-gfp-mreB*	0.15% xylose	(30)
TNVS175	*trpC2 amyE::spc Pxyl-murG-msfgfp*	0.025% xylose	(30)
TNVS284	*trpC2 amyE3': spec-PxylR-mraY-6GS-msfgfp-amyE5'*	0.05% xylose	(30)
JB047	*liaI::pMUTIN4 liaI-lacZ*	-	(31)

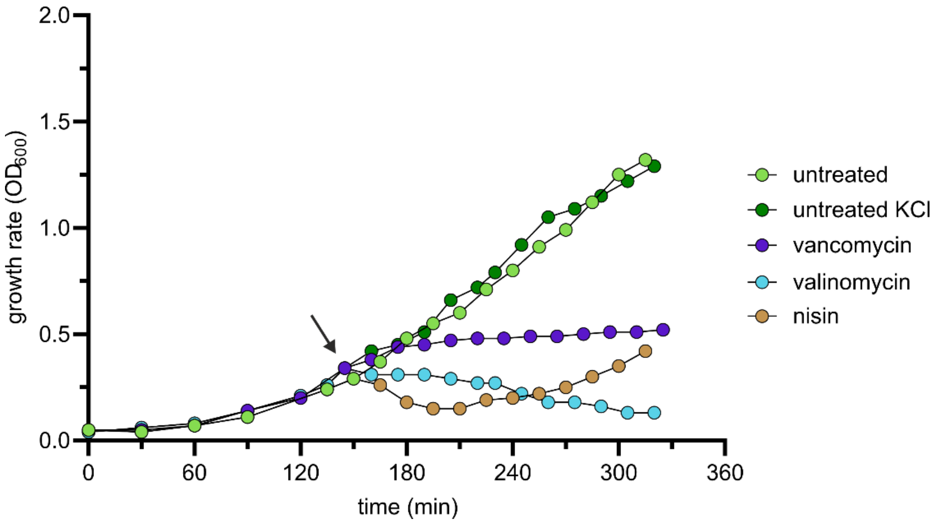

FIG S1 Growth inhibition of *B. subtilis* 168CA by valinomycin, vancomycin, and nisin in MHB at 30 °C. Arrow marks timepoint of antibiotic addition.

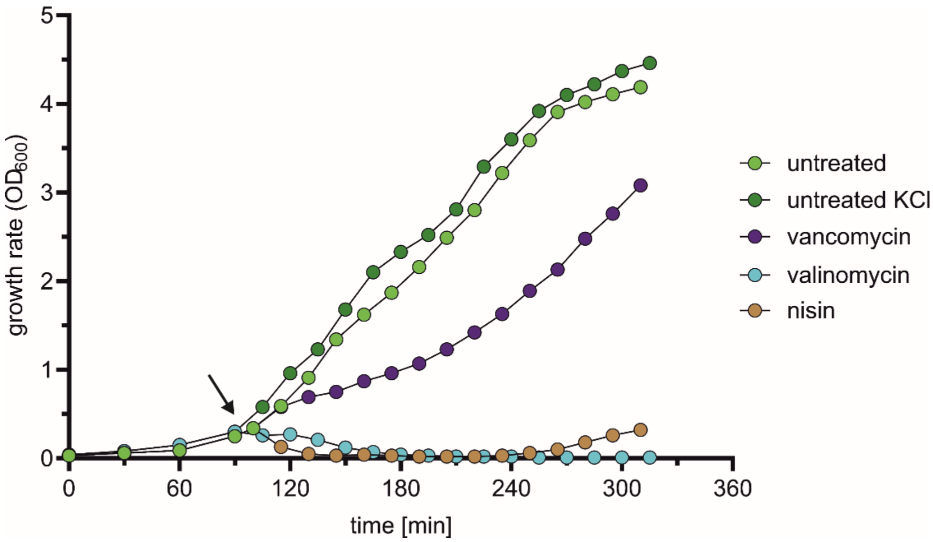

FIG S2 Growth inhibition of *B. subtilis* 168CA by valinomycin, vancomycin, and nisin in LB at 37 °C. Arrow marks timepoint of antibiotic addition.

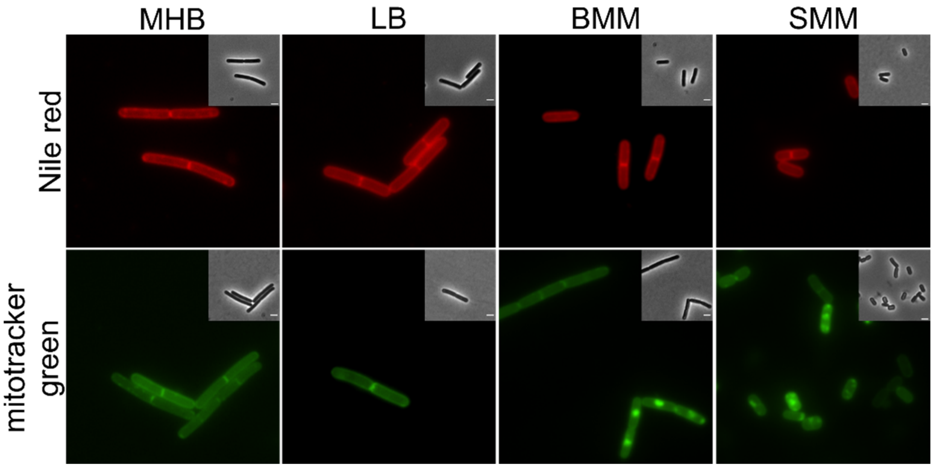

FIG S3 Media compatibility of Nile red and mitotracker green. All cells were grown at 30 °C and stained in mid-exponential growth phase. Scale bars 2 µm.

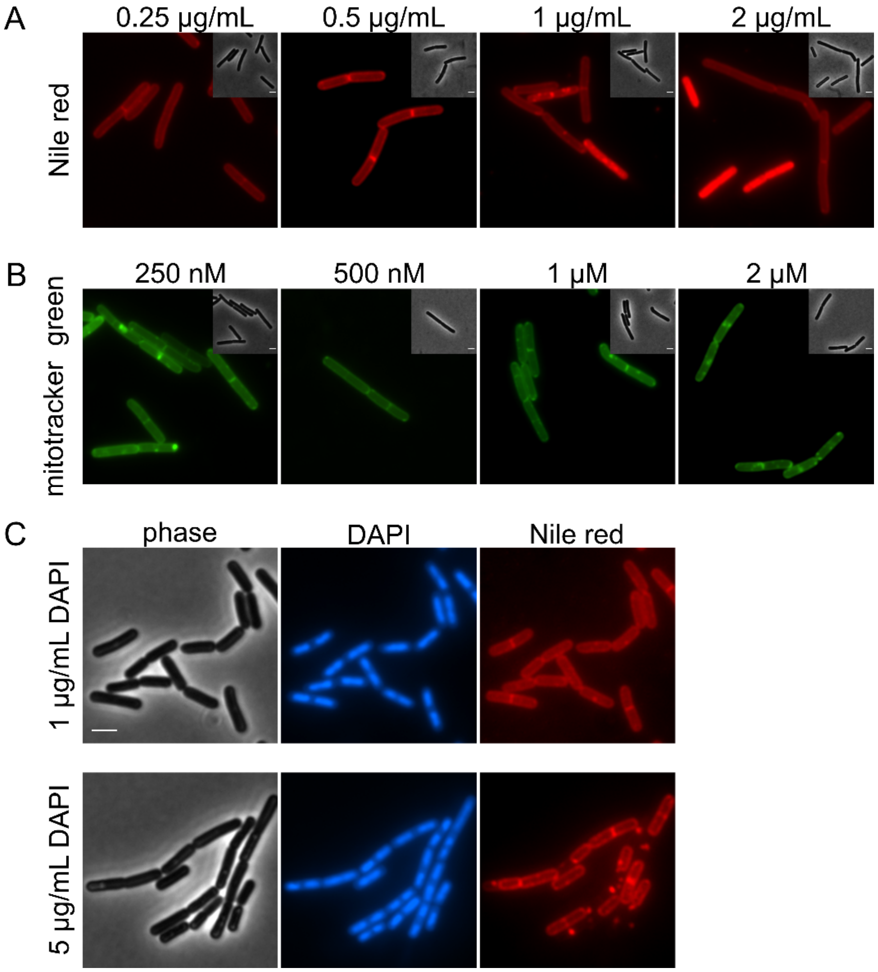

FIG S4 Selection of membrane dye concentration. **(A)** Nile red, **(B)** mitotracker green, **(C)** DAPI. All cells were grown in MHB at 30 °C and stained in mid-exponential growth phase for 5 min prior to microscopy. Scale bars 2 μm. For these conditions, we recommend 0.25-0.5 g/mL Nile red, 0.5-1 μg/mL mitotracker green, and up to 1 μg/mL DAPI.

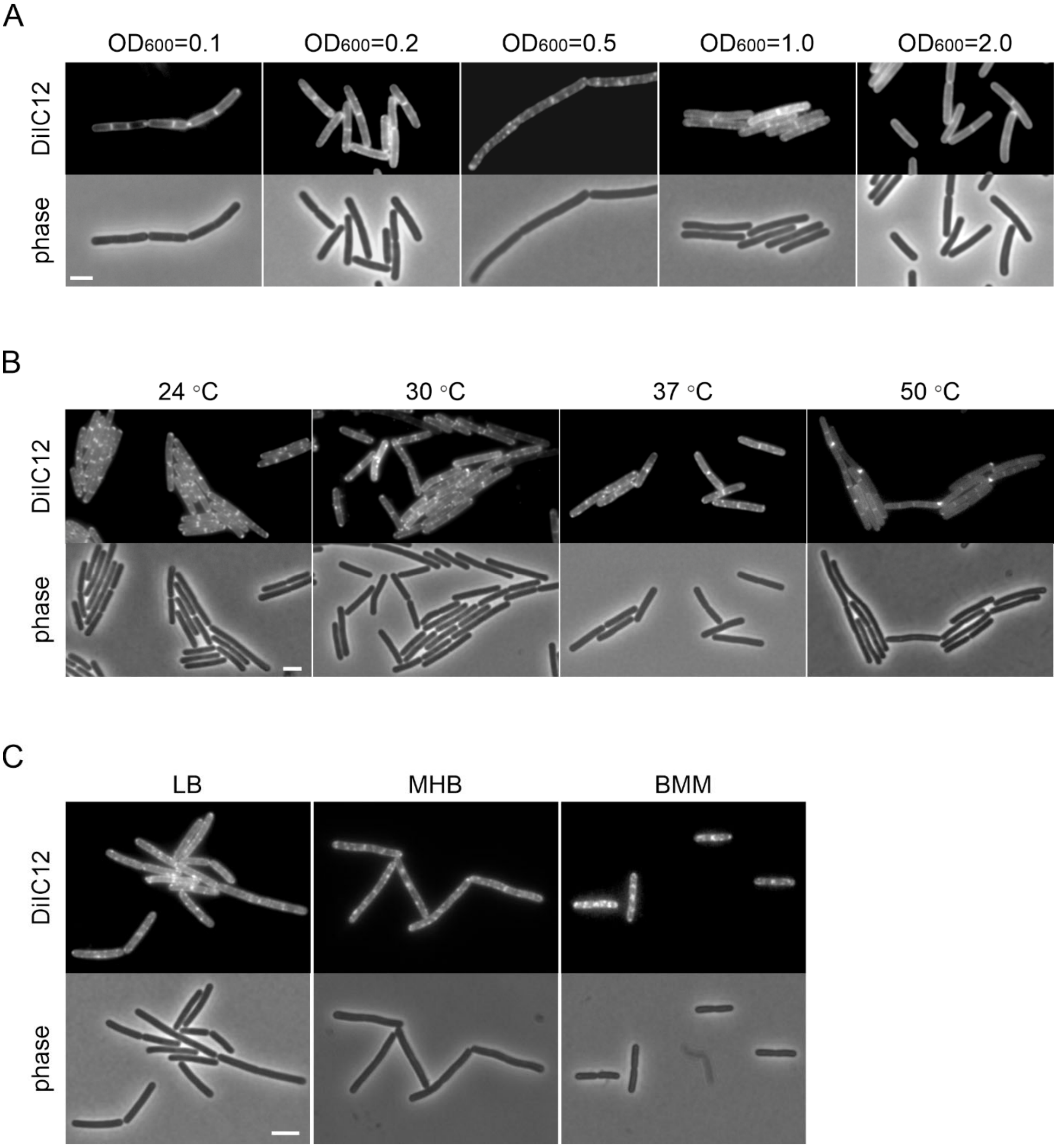

FIG S5 Influence of growth phase, temperature, and medium on DiIC12 staining of *B. subtilis* 168CA. **(A)** RIFs are clearly visible in exponential growth phase. For *B. subtilis* 168CA grown in LB at 30 °C, RIFs can be observed between an OD_{600} of 0.2 and 1.0 with best results obtained at an OD_{600} of 0.5. **(B)** Impact of growth temperature. *B. subtilis* 168 grows in LB at temperatures between ~20 and 50 °C. Regular RIFs are visible at 24, 30, and 37 °C while at 50 °C, where cells can still grow but are considerably stressed, clear clusters are observed. All images were taken at an OD_{600} of 0.3. **(C)** RIFs in different growth media. In the full media LB and MHB cells grow longer and display more and smaller RIFs than in the minimal medium BMM, in which cells grow more slowly. Cultures were grown at 37 °C and images were taken at an OD_{600} of 0.3.

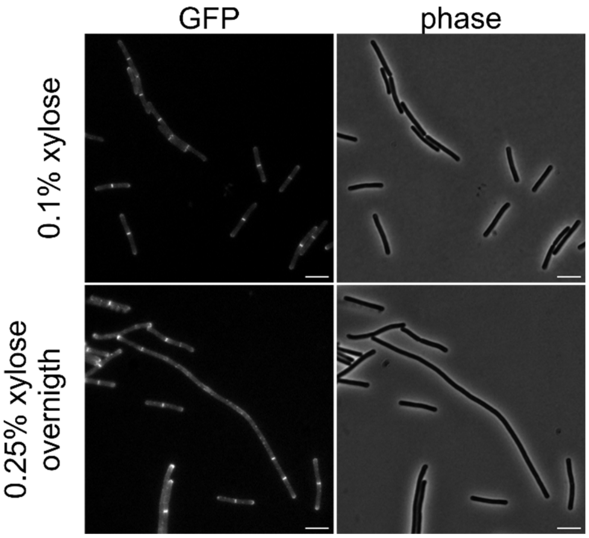

FIG S6 Influence of induction time on MinD localization. Cells were grown in MHB at 30 °C. Xylose was added either when diluting the overnight culture (top) or in both the overnight culture and the diluted culture (bottom). Scale bar 5 µm.

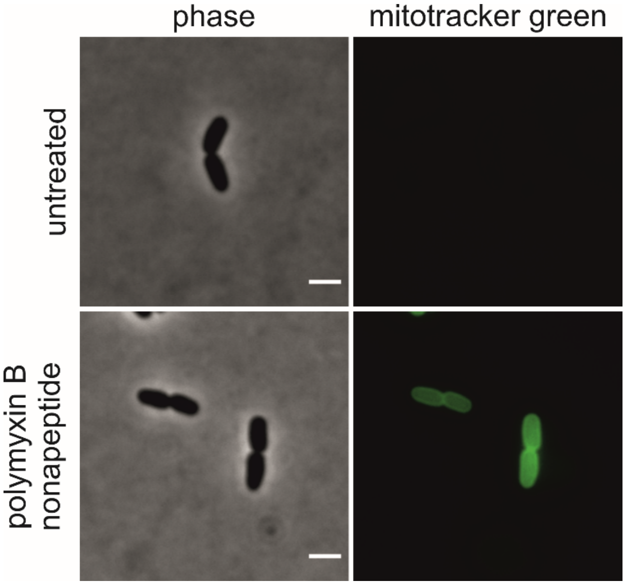

FIG S7 Selective inner membrane staining of outer membrane-permeable *E. coli* cells. *E. coli* MC4100 was grown in MHB at 37 °C. At an OD_{600} of 0.3, cultures were treated with 10 µg/mL polymyxin B nonapeptide for 5 min followed by staining with 4 µM mitotracker green for an additional 5 min. Scale bar 2.5 µm.

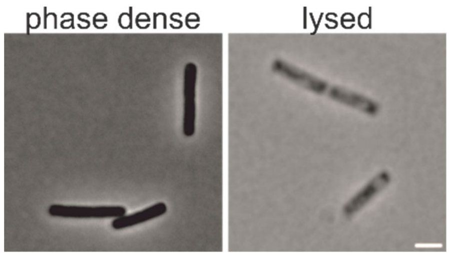

FIG S8 Phase contrast images of healthy, phase-dense cells (left) and lysing cells losing phase contrast (right). Healthy cells appear homogenously dark (=phase dense) with clearly defined cell boundaries, while lysing cells appear as light gray cells with granular texture and undefined boundaries.

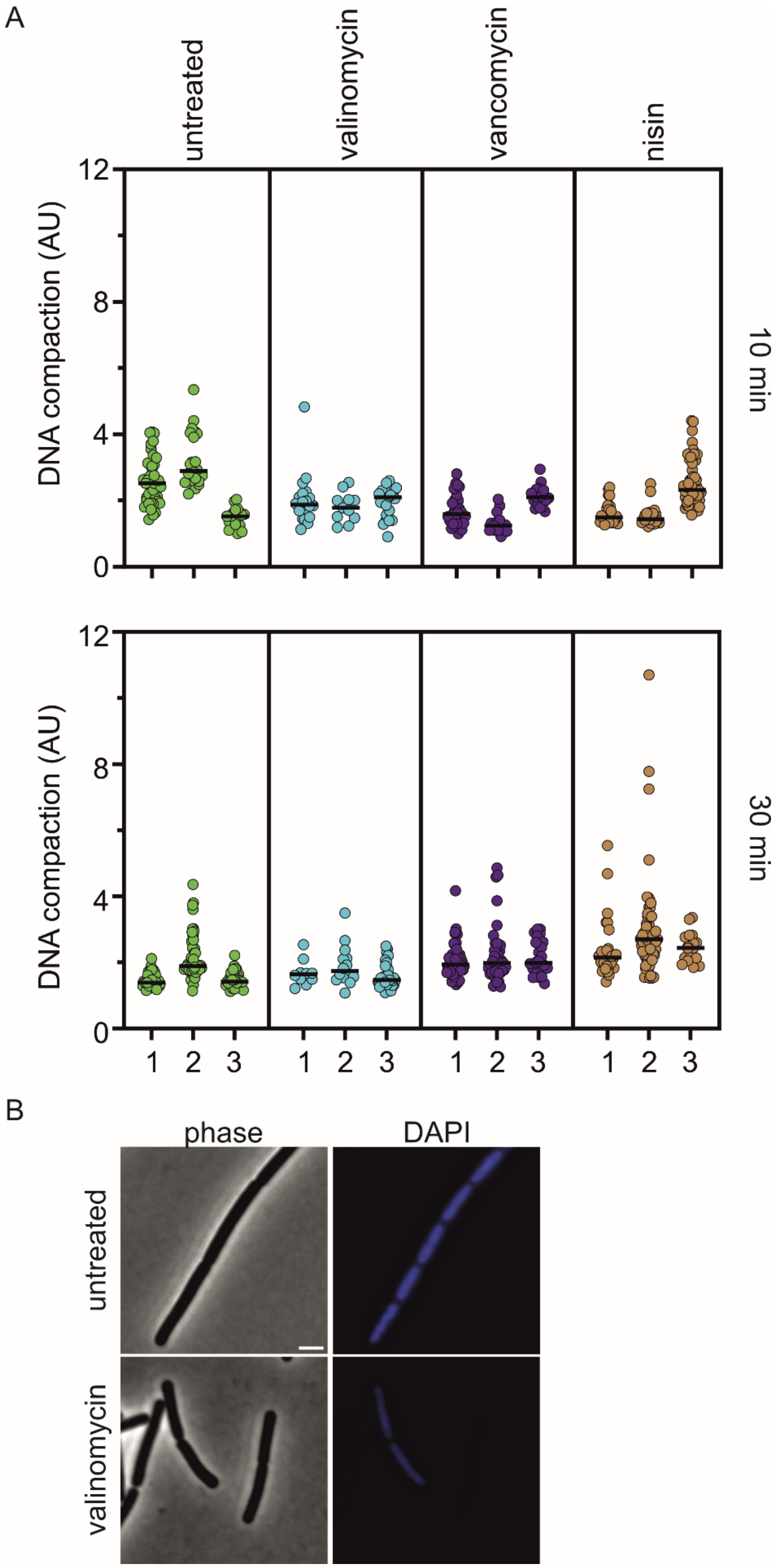

FIG S9 Nucleoid compaction analysis of *B. subtilis* MW54 (*PrpsD-msfGFP*) treated with valinomycin, vancomycin, and nisin. **(A)** Nucleoid compaction values measured with MicrobeJ. Numbers on the x-axis refer to individual biological replicates. **(B)** Example of DNA phenotype that escapes quantification due to insufficient fluorescence intensity.

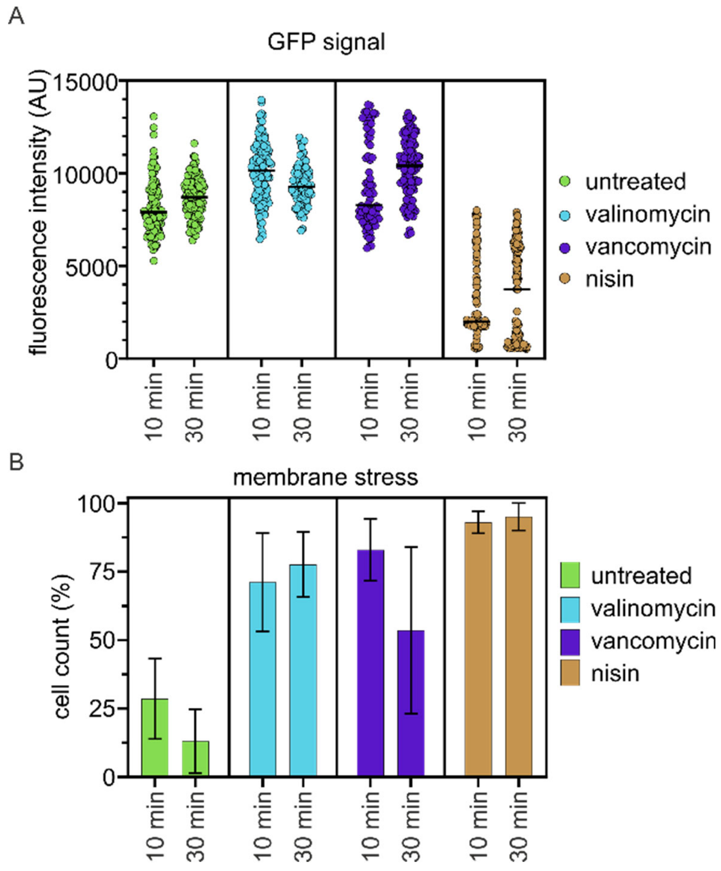

FIG S10 Quantification of intracellular GFP intensity **(A)** and membrane damage **(B)** of *B. subtilis* MW54 (*PrpsD-msfGFP*) treated with valinomycin, vancomycin, and nisin.

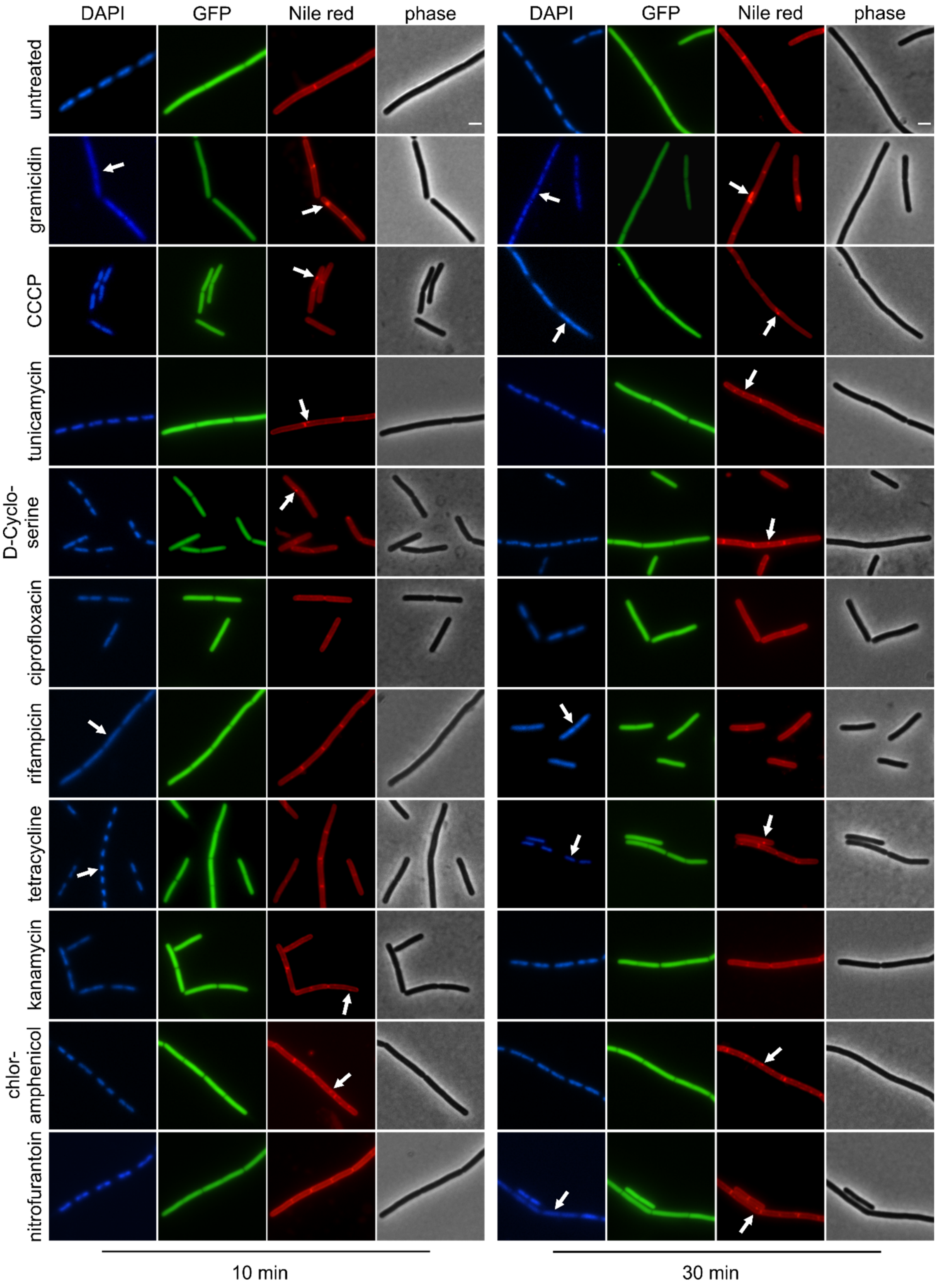

FIG S11 Bacterial cytological profiling of *B. subtilis* bSS82 (*PrpsD-gfp*) treated with antibiotics for 10 and 30 min. DNA was stained with DAPI, cytosolic GFP was expressed from the constitutive *PrpsD* promoter, and membranes were stained with Nile Red. Morphological changes are marked with arrows. Scale bar 2 µm.

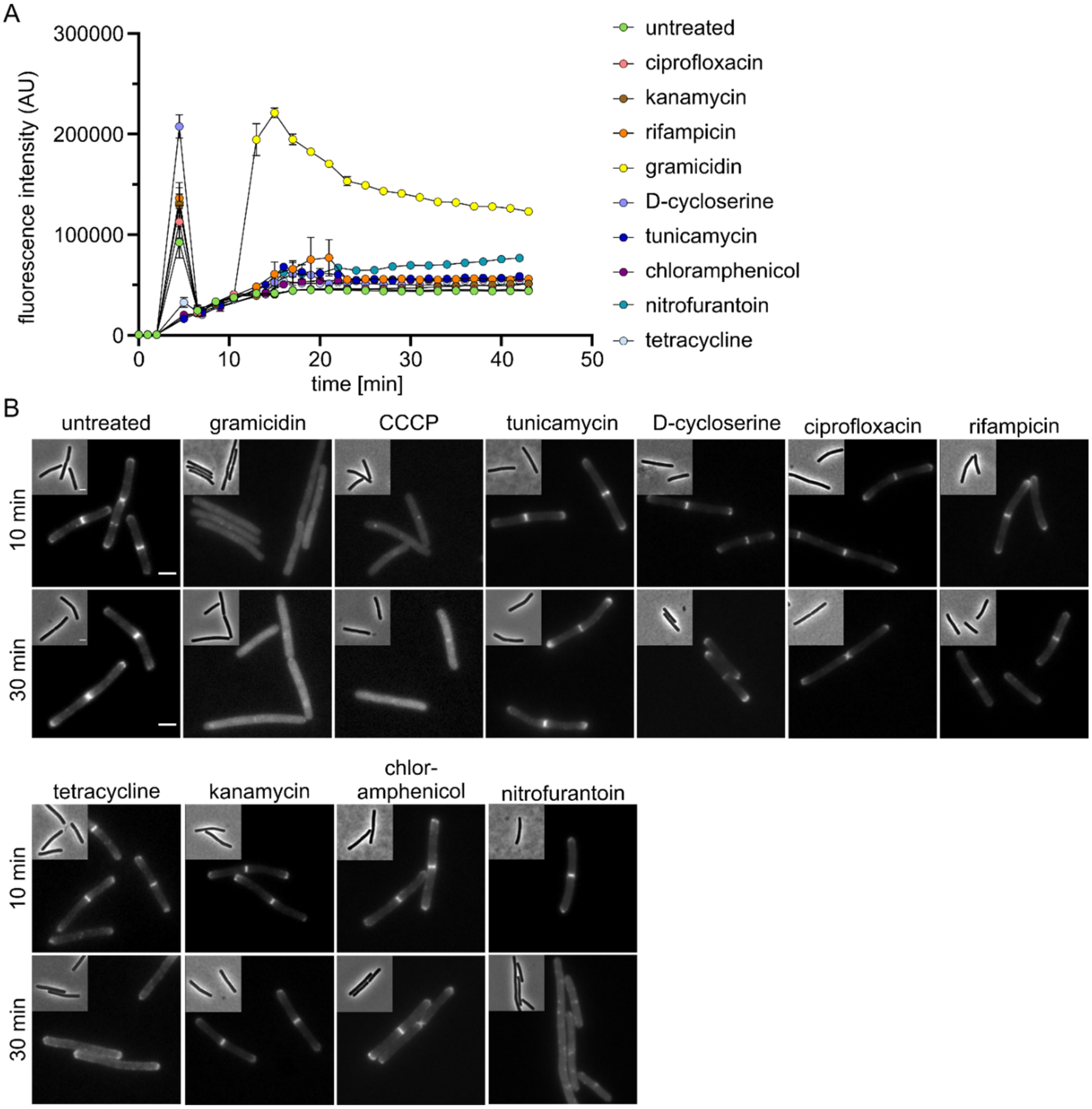

FIG S12 Effects of antibiotics on the membrane potential. **(A)** Spectroscopic membrane potential measurements of *B. subtilis* 168CA (wild type) with the fluorescence dye DiSC$_3$5. Note that CCCP could not be measured due to interference of the compound with the dye. **(B)** Localization of the cell division regulation protein MinD after 10 and 30 min of antibiotic treatment (strain *B. subtilis* TB35 (*Pxyl-gfp-minD*)). Scale bars 2 µm.

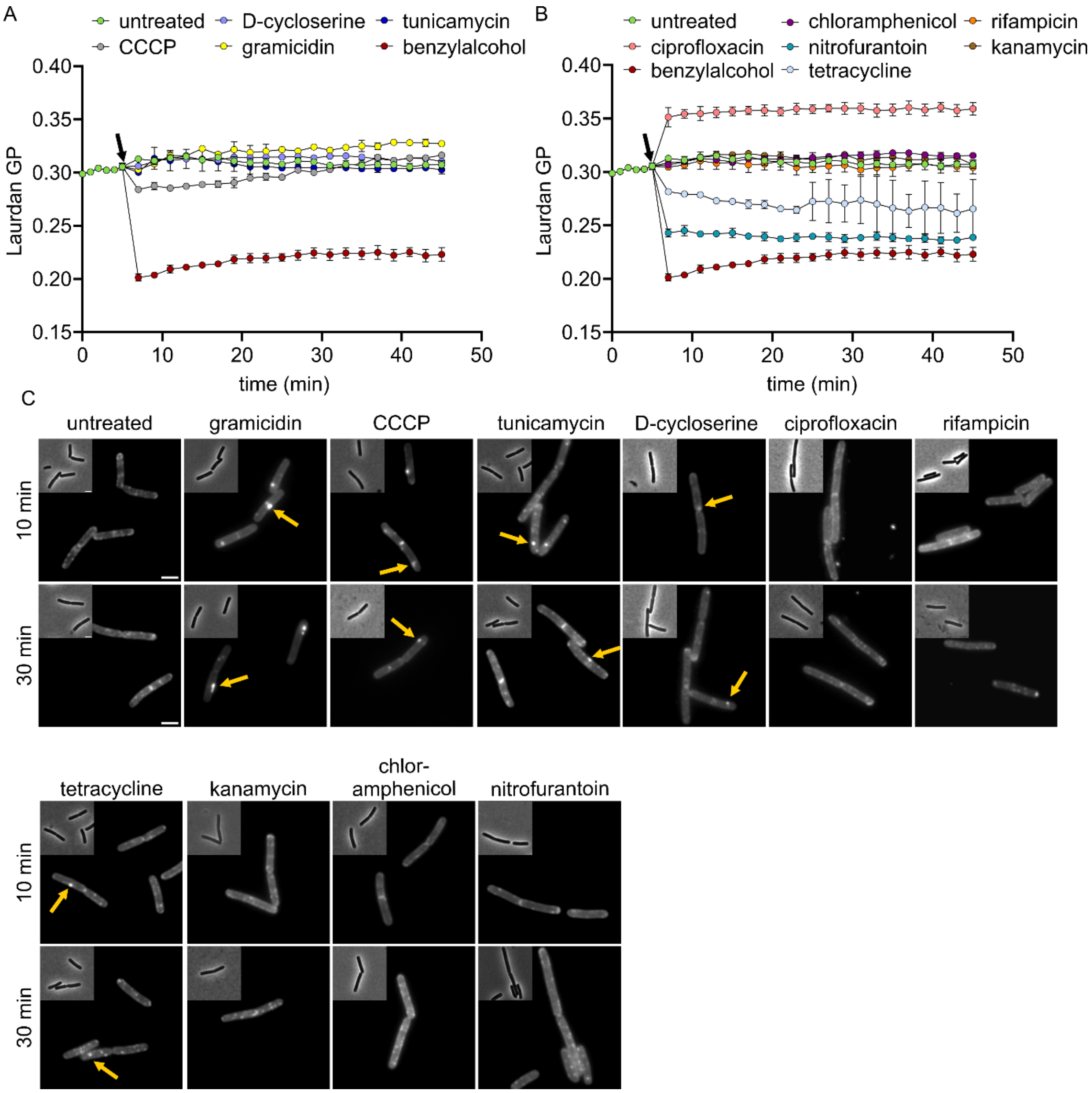

FIG S13 Effects of antibiotics on membrane fluidity. **(A)** Overall membrane fluidity of *B. subtilis* 168CA (wild type) measured by Laurdan generalized polarization (GP). Black arrow indicates timepoint of antibiotic addition. **(B)** Visualization of fluid membrane microdomains (RIFs) in *B. subtilis* 168CA (wild type) with the fluidity-sensitive fluorescence dye DiIC12. Large DiIC12 clusters are indicated with yellow arrows. Scale bars 2 µm.

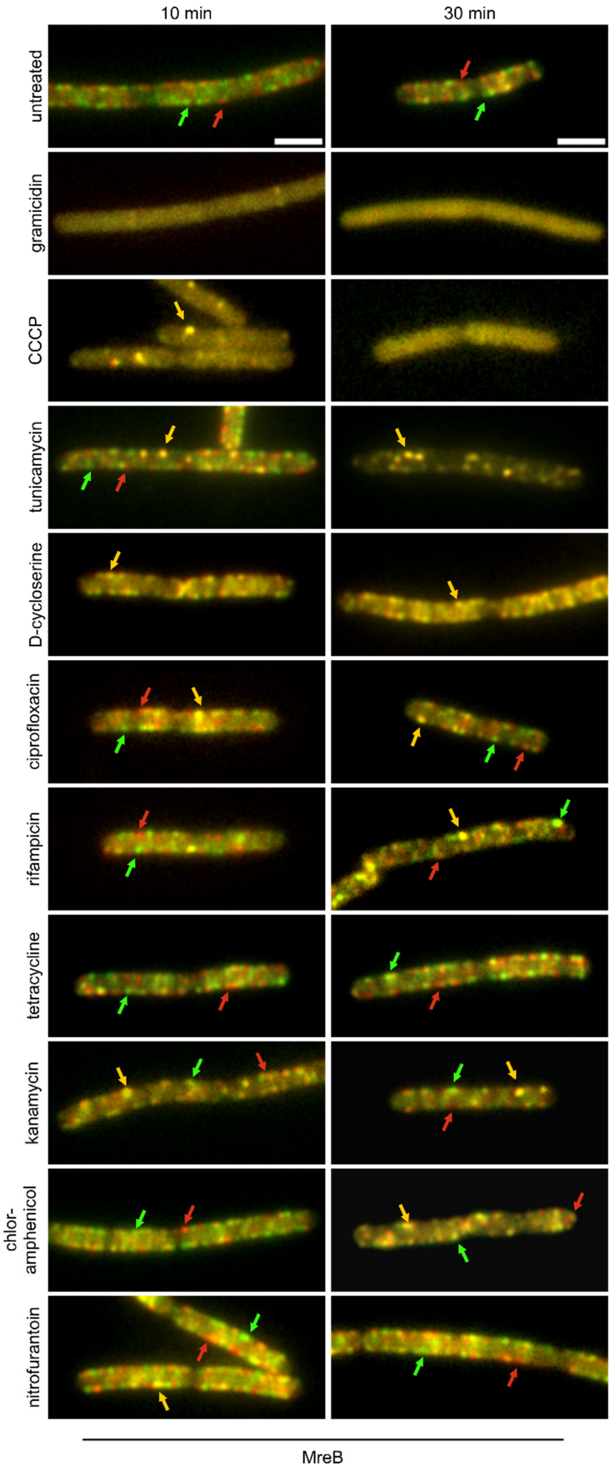

FIG S14 Effects of antibiotics on MreB mobility. MreB mobility was assessed by recording two separate images of the same *B. subtilis* MW10 (*Pxyl-gfp-mreB*) cells in a 30-sec interval. Individual images were false-colored red and green and overlaid, resulting in perfect overlap when MreB movement is stalled (yellow foci), and separate green and red foci when it is retained. Exemplary static foci are indicated by yellow arrows, while exemplary distinct red and green foci are indicated by arrows in the corresponding colors. Scale bar 2 µm.

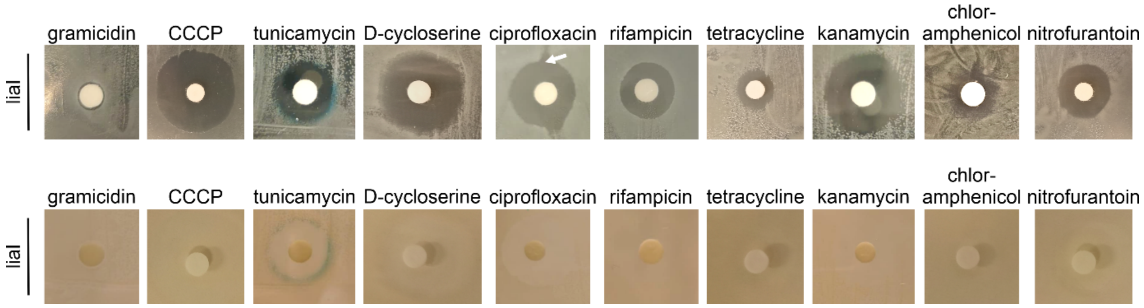

FIG S15 Effects of antibiotics on *PliaI* induction. Induction of *PliaI* in JB047 (*liaI-lacZ*) was tested in a disk diffusion assay on agar plates containing X-gal. A blue ring around the inhibition zone indicates activation of the *PliaI* promoter. The top row shows pictures taken against a dark background to visualize the inhibition zone, while the bottom row shows the same samples photographed against a light background to visualize the blue color.

References

1. Kamal El-sagheir AM, Abdelmesseh Nekhala I, Abd El-Gaber MK, Aboraia AS, Persson J, Schäfer A-B, Wenzel M, Omar FA. 2023. N4-Substituted Piperazinyl Norfloxacin Derivatives with Broad-Spectrum Activity and Multiple Mechanisms on Gyrase, Topoisomerase IV, and Bacterial Cell Wall Synthesis. ACS Bio Med Chem Au 3:494-506.

2. Wenzel M, Bandow JE. 2011. Proteomic signatures in antibiotic research. Proteomics 11:3256–3268.

3. Wenzel M, Kohl B, Münch D, Raatschen N, Albada HB, Hamoen L, Metzler-Nolte N, Sahl HG, Bandow JE. 2012. Proteomic response of *Bacillus subtilis* to lantibiotics reflects differences in interaction with the cytoplasmic membrane. Antimicrob Agents Chemother 56:5749–5757.

4. Hoeprich PD, Finn PD. 1971. Influence of culture media on the antistaphylococcal activity of fosfomycin. Appl Microbiol 22:781–783.

5. Rewak-Soroczynska J, Dorotkiewicz-Jach A, Drulis-Kawa Z, Wiglusz RJ. 2022. Culture Media Composition Influences the Antibacterial Effect of Silver, Cupric, and Zinc Ions against Pseudomonas aeruginosa. Biomolecules 12:963.

6. Schäfer AB, Wenzel M. 2020. A How-To Guide for Mode of Action Analysis of Antimicrobial Peptides. Front Cell Infect Microbiol 10:540898.

7. Saeloh D, Tipmanee V, Jim KK, Dekker MP, Bitter W, Voravuthikunchai SP, Wenzel M, Hamoen LW. 2018. The novel antibiotic rhodomyrtone traps membrane proteins in vesicles with increased fluidity. PLoS Pathog 14:e1006876.

8. Wenzel M, Vischer NOE, Strahl H, Hamoen LW. 2018. Assessing membrane fluidity and visualizing fluid membrane domains in bacteria using fluorescent membrane dyes. Bio-protocol 8:e3063.

9. Schäfer A-B, Steenhuis M, Jim KK, Neef J, O'Keefe S, Whitehead RC, Swanton E, Wang B, Halbedel S, High S, van Dijl JM, Luirink J, Wenzel M. 2023. Dual Action of Eeyarestatin 24 on Sec-Dependent Protein Secretion and Bacterial DNA. ACS Infect Dis 9:253–269.

10. Jonkers TJH, Steenhuis M, Schalkwijk L, Luirink J, Bald D, Houtman CJ, Kool J,

Lamoree MH, Hamers T. 2020. Development of a high-throughput bioassay for screening of antibiotics in aquatic environmental samples. Sci Total Environ 729:139028.

11. Buttress JA, Halte M, Te Winkel JD, Erhardt M, Popp PF, Strahl H. 2022. A guide for membrane potential measurements in Gram-negative bacteria using voltage-sensitive dyes. Microbiology 168:001227.

12. Kamal El-sagheir AM, Abdelmesseh Nekhala I, Abd El-Gaber MK, Aboraia AS, Persson J, Schäfer A-B, Wenzel M, Omar FA. 2023. Rational design, synthesis, molecular modeling, biological activity, and mechanism of action of polypharmacological norfloxacin hydroxamic acid derivatives. RSC Med Chem 14:2593-2610.

13. Kamal El-Sagheir AM, Abdelmesseh Nekhala I, Abd El-Gaber MK, Aboraia AS, Persson J, Schäfer A-B, Wenzel M, Omar FA. 2023. Design, Synthesis, Molecular Modeling, Biological Activity, and Mechanism of Action of Novel Amino Acid Derivatives of Norfloxacin. ACS Omega 8:43271–43284.

14. Liu X, Meiresonne NY, Bouhss A, den Blaauwen T. 2018. FtsW activity and lipid II synthesis are required for recruitment of MurJ to midcell during cell division in *Escherichia coli*. Mol Microbiol 109:855–884.

15. Schindelin J, Arganda-Carreras I, Frise E, Kaynig V, Longair M, Pietzsch T, Preibisch S, Rueden C, Saalfeld S, Schmid B, Tinevez JY, White DJ, Hartenstein V, Eliceiri K, Tomancak P, Cardona A. 2012. Fiji: An open-source platform for biological-image analysis. Nat Methods 9:676–682.

16. Schneider CA, Rasband WS, Eliceiri KW. 2012. NIH Image to ImageJ: 25 years of image analysis. Nat Methods 9:671-675.

17. Syvertsson S, Vischer NOE, Gao Y, Hamoen LW. 2016. When phase contrast fails: ChainTracer and NucTracer, two ImageJ methods for semi-automated single cell analysis using membrane or DNA staining. PLoS One 11:1–11.

18. Ducret A, Quardokus EM, Brun Y V. 2016. MicrobeJ, a tool for high throughput bacterial cell detection and quantitative analysis. Nat Microbiol 1:1–7.

19. Wenzel M, Dekker MP, Wang B, Burggraaf MJ, Bitter W, van Weering JRT, Hamoen

LW. 2021. A flat embedding method for transmission electron microscopy reveals an unknown mechanism of tetracycline. Commun Biol 4:306.

20. Ojcius DM, Zychlinsky A, Zheng LM, Young JD-E. 1991. Ionophore-induced apoptosis: Role of DNA fragmentation and calcium fluxes. Exp Cell Res 197:43–49.

21. Vischer NOE, Verheul J, Postma M, van den Berg van Saparoea B, Galli E, Natale P, Gerdes K, Luirink J, Vollmer W, Vicente M, den Blaauwen T. 2015. Cell age dependent concentration of Escherichia coli divisome proteins analyzed with ImageJ and ObjectJ. Front Microbiol 6:586.

22. Strahl H, Hamoen LW. 2010. Membrane potential is important for bacterial cell division. Proc Natl Acad Sci U S A 107:12281–12286.

23. te Winkel JD, Gray DA, Seistrup KH, Hamoen LW, Strahl H. 2016. Analysis of antimicrobial-triggered membrane depolarisation using voltage sensitive dyes. Front Cell Dev Biol 4:29.

24. Humphrey M, Abdelmesseh Nekhala I, Scheinpflug K, Krylova O, Schäfer A-B, Buttress JA, Wenzel M, Strahl H. 2023. Tracking Global and Local Changes in Membrane Fluidity Through Fluorescence Spectroscopy and Microscopy, p. 203–229. *In* Sass, P (ed.), Methods in Molecular Biology.

25. Scheinpflug K, Krylova O, Strahl H. 2017. Measurement of Cell Membrane Fluidity by Laurdan GP: Fluorescence Spectroscopy and Microscopy. Methods Mol Biol 1520:159–174.

26. Wenzel M, Rautenbach M, Vosloo JA, Siersma T, Aisenbrey CHM, Zaitseva E, Laubscher WE, van Rensburg W, Behrends J, Bechinger B, Hamoen LW. 2018. The multifaceted antibacterial mechanisms of the pioneering peptide antibiotics tyrocidine and gramicidin S. MBio 9:e00802-e00818.

27. Anagnostopoulos C, Spizizen J. 1960. Requirements for transformation in *Bacillus subtilis*. J Bacteriol 81:741–746.

28. Jahn N, Brantl S, Strahl H. 2015. Against the mainstream: the membrane-associated type I toxin BsrG from *Bacillus subtilis* interferes with cell envelope biosynthesis without increasing membrane permeability. Mol Microbiol 98:651–666.

29. Marston AL, Thomaides HB, Edwards DH, Sharpe ME, Errington J. 1998. Polar

localization of the MinD protein of *Bacillus subtilis* and its role in selection of the mid-cell division site. Genes Dev 12:3419–3430.

30. Müller A, Wenzel M, Strahl H, Grein F, Saaki TN V, Kohl B, Siersma T, Bandow JE, Sahl H-G, Schneider T, Hamoen LW. 2016. Daptomycin inhibits cell envelope synthesis by interfering with fluid membrane microdomains. Proc Natl Acad Sci U S A 113:E7077–E7086.

31. Mascher T, Zimmer SL, Smith T-A, Helmann JD. 2004. Antibiotic-Inducible Promoter Regulated by the Cell Envelope Stress-Sensing Two-Component System LiaRS of *Bacillus subtilis*. Antimicrob Agents Chemother 48:2888–2896.
